# Efficacy of Chloroquine for the Treatment of *Vivax malaria* in Northwest Ethiopia

**DOI:** 10.1371/journal.pone.0161483

**Published:** 2016-08-31

**Authors:** Habtamu Bedimo Beyene, Melkamu Bedimo Beyene, Yehenew Asmamaw Ebstie, Zelalem Desalegn

**Affiliations:** 1 Department of Microbiology, Immunology and Parasitology, College of Health Sciences, Addis Ababa University, Addis Ababa, Ethiopia; 2 Department of Public Health, College of Health Sciences, Bahir Dar University, Bahir Dar, Ethiopia; Agency for Science, Technology and Research - Singapore Immunology Network, SINGAPORE

## Abstract

**Background:**

Resistance to anti-malarials is a major challenge for effective malaria control in sub-Saharan Africa. This triggered a need for routine monitoring of the efficacy of the antimalarial drugs every two years in all malaria endemic countries. Chloroquine remained the drug of choice for the treatment of vivax malaria in Ethiopia. Though, a strong scientific evidence of chloroquine resistance to *P*.*vivax* that could have brought change of treatment regimen is yet to be established in Ethiopia, continuous and regular monitoring of drug’s efficacy is critical for establishing rational anti-malarial drug policies. This study therefore, assessed the therapeutic efficacy of Chloroquine (CQ) for the treatment of *Plasmodium vivax* infections in Northwestern Ethiopia.

**Methods:**

An observational, 28- day therapeutic clinical efficacy study was conducted from August to December, 2014, in Northwest Ethiopia. Patients confirmed to have monoinfection of vivax malaria, aged above 6 months were included. All subjects were treated with standard chloroquine dose of 25 mg/kg for three (3) days. Parasitological and clinical outcomes of treated patients were then evaluated on days 1, 2, 3, 7, 14, 21, and 28 during the entire 28-day follow-up period. A portable spectrophotometer (HemoCue Hb 301 System, Sweden) was used to estimate hemoglobin concentration.

**Results:**

A total of 69 subjects had completed follow up. Some 57/69 (82.6%) had fever at enrolment and the rest 12 patients 48 hours before enrollment. Out of total, 65/69 (94.2%) and 66/69 (95.6%) of the study subjects were free of fever by day 1 and day 2 respectively but fever was cleared in all subjects by day 3. At base line the mean asexual parasitemia was 3540 parasites/μL of blood. Parasite carriage on day 3 was 3%. The overall cure rate (an adequate and clinical parasitological response) was very high (97%) [(95% CI = 93.1–99.4)]. The time to parasite, fever and gametocyte clearance as expressed in mean (SD) was 35 (3), 25 (4.6), 28 (3.2) hours respectively. Mean hemoglobin was significantly increased (P<0.001) from 12.2 (7–15) g/dl at day 0 to 13.3 (10–16) g/dl on day 28.

**Conclusions:**

In view of our findings, CQ remains efficacious for the treatment of vivax malaria in the study area. However, there is a need to monitor CQR regularly using molecular and or biochemical tools for better evaluation of treatment outcomes.

## Introduction

There are five species of plasmodia known to infect humans and cause malaria of which, *Plasmodium vivax* is the most widely distributed species responsible for 25–40% of malaria cases worldwide [1]. A considerable morbidity associated with *P*.*vivax* has been reported in many studies [2–4]. In Ethiopia, vivax malaria accounts for approximately 40% of all malaria cases, and most recently it seems, this proportion is even rising. However, not enough attention has been put towards vivax malaria operational research and its control activities, due to the notion that it is less deadly than falciparum malaria [2,5,6].

Chloroquine (CQ) and primaquine (PQ) are drugs of choice in current use for the treatment of vivax malaria. Primaquine being the only effective drug against the hypnozoite stage. In areas where susceptibility to CQ is ascertained, the recommended doses is CQ 25 mg/kg bw (body weight) during three consecutive days (10, 10 and 5 mg/kg bw) and Primaquine (PQ) at 0.25–0.75 mg/kg bw during 14 days [7–9] Of course, the 14-day treatment effectively cures primary blood infections and is the most effective for preventing relapses [10]. However, in areas where CQR is substantial, artemisinin combination therapy (ACT) is recommended, also in combination with PQ for radical cure [9].

The first evidence of CQ resistant (CQR) to *P*. *vivax* began to emerge in 1989 [11,12], 30 years after the documentation of CQR *Plasmodium falciparum*. Since, then the problem of CQR to *plasmodium vivax* continued to occur in different regions at varying degrees. For instance, in Indonesia, East Timor and Papua New Guinea, CQ-resistant vivax malaria has already reached an alarming prevalence [13,14]. CQR has also occurred in Latin American countries such as (Guyana, Peru and Brazil) [15]. The four clinical trials carried out in Asia (Thailand and Pakistan) and Africa (Ethiopia), for instance, showed that CQ alone (25 mg/kg over 3 days) is less effective against *P*. *vivax* asexual blood stages than CQ (25 mg/kg over 3 days) co-administered with PQ (15 mg of PQ base/day for 14 days) over 28 days [10].

In Ethiopia, Chloroquine 150 mg base tablet *or* chloroquine syrup 50 mg base is the first line drug of choice for the treatment of vivax malaria. The ideal dose is 10 mg base/kg po immediately (Day 1), followed by 10 mg base/kg po at 24 hours (Day 2), and 5mg base/kg po at 48 hours (Day 3) for a total dose of 25 mg chloroquine base/kg over three days with a maximum total of 1,500 mg chloroquine base (maximum of 2,500 mg chloroquine phosphate salt) over three days in three divided doses [16].

Sulphadoxine-pyrimethamine (SP) replaced CQ triple-dose (25 mg base/kg) for the treatment of *P*.*falciparum* and mixed infections of vivax and falciparum malaria in 1998. Due to persisted *P*.*falciparum* resistance to SP [17–19] however, CQ has been in use for the treatment of both *P*.*falciparum* and *P*.*vivax* until failure was documented in 1996 [19]. Following this, Artemether-lumefantrine (AL) (Coartem®) replaced SP in 2004 as a first-line drug to treat uncomplicated *P*. *falciparum* and mixed infection of *P*. *falciparum and P*.*vivax* malaria in Ethiopia. But, CQ continued to be the first line drug for the treatment of *P*.*vivax* monoinfection, in Ethiopia and the study area.

The emergence of resistance to anti malarials threatens efforts towards malaria control and elimination. Resistance to Drugs has been implicated in the spread of malaria to new areas and re-emergence of malaria in areas where the disease had been eliminated. It also plays significant role in the occurrence and severity of epidemics in some parts of the world [20] and results in an ongoing diseases transmission. Therefore, there is a need to monitor, the status of drug resistance to anti-malarials on continuous and regular basis (often at 2 years interval. To do so, three main tools are used for drug resistance monitoring and assessing including therapeutic efficacy tests (*In* vivo), in vitro tests, and analyses of molecular markers.

Therapeutic efficacy assessment according to the standard protocol of the WHO is the most useful and cost effective tool for updating national treatment policies. These include, therapeutic assessment based on evaluation of the clinical and parasitological outcomes remains the mainstay of monitoring the efficacies of anti-malarial regimens and is recommended [21]. Measures such as parasite clearance time, fever clearance time or gametocyte clearance time in vivo and in vitro assays are used to indirectly detect any variation in parasite sensitivity thereby facilitating early warning in case of emergence of tolerance or resistance [22, 23].

Although CQ has been the cheapest and mainstay, treatment for vivax, malaria in Ethiopia, its efficacy is not well established, throughout the country. There is, little information on the response of *P*. *vivax* to CQ in Ethiopia. A study conducted in Debre Zeit area in 1996 showed a 2% parasitological failure to CQ treatment on day 7 [24] and more recently an increased overall failure rate of 5.76% at same area [25] was documented. A 13% failure rate was also observed by Ketema and colleagues in 2009 [26]. Moreover a 3.3% failure rate at Hossana, Sothern Ethiopia [27] was reported in 2014, but this report failed to identify the presence or absence of mixed infections.

More recently, a study conducted in four sites in Southern Ethiopia showed an overall risk of recurrence to be as high as 9.4% and 21.9% in one of the study sites [28]. Such growing evidences of the occurrence of treatment failure rates at varying degrees in Ethiopia necessitated a need for further assessment of the drug’s treatment outcome in different setting to help support policy makers if changes in treatment policies are to be made. Though, strong scientific evidence towards chloroquine resistance to *P*.*vivax* that could have brought change of treatment regimen is yet to be established in Ethiopia, continuous and regular monitoring of drug’s efficacy is critical for establishing rational anti-malarial drug policies. There was no similar study done in the present study area as well. Therefore, in the present study, we assessed the efficacy of standard CQ treatment for *P*. *vivax* malaria in an open one arm observational, prospective follow up study in patients presented to the outpatient department of Bullen Health Center, Northern Ethiopia.

## Materials and Methods

### Study area

The Benishangul Gumuz Regional State (BFGS) has an estimated area of 51,000 square kilometers and shares common borders with the State of Amhara in the east, the Sudan in the north-east, and the State of Oromia in the south. It is divided into 3 administrative zones, 19 Weredas and 33 Kebeles (the smallest administrative units) [29]. Metekel is the largest zone with an area of 26,272 square kilometers followed by Assosa and Kamashi. The state has diverse topography and climate. The later includes the familiar traditional zones—"kola", "dega", and "woyna dega". About 75% of the State is classified as "kola" (low lands), which is below 1500 meters above sea level. The altitude ranges from 550 to 2,500 meters above sea level. The average annual temperature reaches from 20-250C. During the hottest months (January—May) it reaches a 28–34°C. Bullen is one of the towns in the Metekel zone of BGRS, which lies on a longitude of 10°0o0°N 39°590 E36°0'0"E and latitude of 12°0'0"N. The town has a total of 30,828 inhabitants, and malaria is implicated to be one of the major health problem in the area [30].

Both *P*.*vivax* and *P*.*falciparum* are dominant endemic species in the study area accounting for 13.3% and 86.7% respectively [31]. Though, malaria transmission dynamics is highly seasonal and unstable generally in Ethiopia, with transmission peaking from September to December and from April to June every year [32], information is lacking on attributes of vivax malaria such as relapsing pattern and its intensity of transmission in this area.

### Study Design and patient recruitment

**Fig 1:**
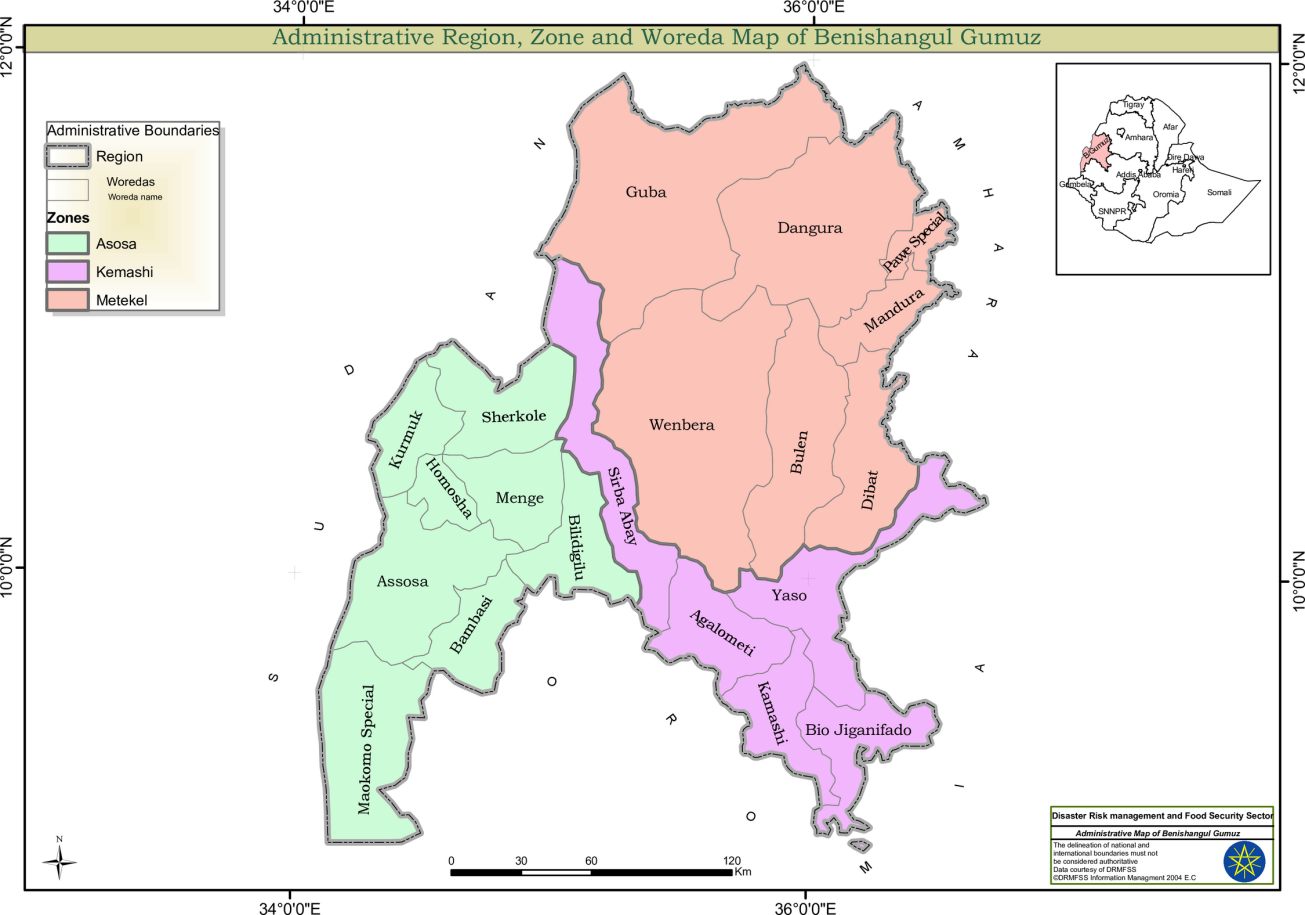
Map of the study site. Metekel Zone, Bullen District.

A one arm prospective, observational follow up study was conducted in Benishangul Gumuz region (BGRS), Metekel Zone, Bullen district, located in Northern-West Ethiopia (Fig 1) in Northwest Ethiopia. The study subjects were recruited from those patients visiting Bullen health centre from 17, August to 19 December, 2014, presented with clinically suspected malaria. Participants with suspected malaria symptoms were initially screened if they had to be included in the study. Patients were enrolled in to follow up only after informed written consent was obtained and if the following criteria were met 1) age > 6 months old, 2) *P*. *vivax* monoinfection with parasitaemia > 250 asexual parasites/μL, 3) presence of fever (axillary temperature ≥ 37.5°C) or history of fever 48 h preceding medical consultation, and 4) ability to swallow oral medication [33]. Patients with signs of severe or complicated symptoms of *vivax* malaria (such as coma, impaired consciousness, respiratory distress, convulsions) [4], mixed *Plasmodium* species, severe malnutrition, fever due to concomitant diseases, prior anti malarial intake during 2 weeks prior to enrollment), pregnant women, and history of allergic reaction to chloroquine were excluded.

### Sample size

A minimal sample size of 69 eligible patients was calculated based on the WHO revised protocol (S1 file) for antimalarial drug efficacy surveillance [33] and prevalence of 3.6% treatment failure rate reported in Southwestern Ethiopia [34], 5% precision, 95% confidence level and 25% contingency (expected loss to follow-up rate and withdrawal of consent during the study.

### Drug administration

In this 28- day follow up study, patients were treated with CQ (Aralen®, Sanofi-Aventis, US) according to the National treatment guidelines with standard dose of 25 mg/kg CQ-sulfate administered for three consecutive days (on day 0 (10 mg base/kg body weight), day 1 (10 mg base/kg body weight), and day 2 (5 mg base/kg body weight) [35, 36] at Bullen health Centre. The 28-day follow-up of CQ efficacy testing was done according to the methods recommended by WHO, the Ethiopian Ministry of Health [35, 37] and the Ethiopian Drug Administration and Control Authority (DACA) [38]. The treatment was administered under direct observation. Patients were followed for 30 minutes post treatment if vomiting occurred. When vomiting take place, the patient was treated with a same full dose of drug. However, patients who vomited twice were excluded, and referred to Pawe Hospital (nearest hospital to the health center) for further management. Eligible subjects were given a personal identification number and received treatment after the study was fully explained and informed consent provided.

### Treatment follow up

The follow-up included a fixed schedule on (day 1, 2, 3, 7, 14, 21, and 28) and with corresponding clinical and laboratory examinations. Assessment and monitoring of parasitological and clinical outcome was made to each patient until day 28. Day 0 was defined as the day a patient was enrolled and received the first dose of CQ. The study subjects were asked to come back to health center on days 1, 2, 3, 7, 14, 21, and 28 and or if experienced malaria episodes before next appointment date and or showed any sign of danger (as unable to drink or breastfed (if child), vomiting, presenting with convulsions, lethargic or unconscious, unable to sit or stand, difficult breathing) [39]. Auxiliary temperature, body weight and clinical conditions were recorded during the follow up period. Health extension workers assisted in visiting and finding a patient/s who did not come to the health care centre on the day of appointment to assure complete the follow-up. But, patients were labeled as lost to follow-up whenever they did not come to the clinic as scheduled and or became inaccessible. Adverse events were monitored and registered at every follow up day.

### Laboratory investigation

For each follow-up day, a capillary blood was collected; both thick and thin blood smears (each in duplicate) were prepared for each patient. Blood smears were stained with 10% Giemsa for 10 min. Diagnosis of malaria parasites was established by using microscopic method and read by two independent laboratory technologists. Discordant results between the two microscopists in species diagnosis were re-examined by a third, independent microscopist, and parasite density was calculated by averaging the two closest counts. A blood film was considered negative when no parasite was found after examining100 fields containing 100 WBCs. Parasite count was made from thick blood smears in relation to the number of white blood cells (WBCs). The number of asexual stages of *P*. *vivax* was counted against 200 WBCs and parasitaemia was determined as follows, based on the standard counting method. No. parasites per/μL = Parasite count × 8,000/200WBC. Hemoglobin level was estimated at two points (days 0 and 28) during the entire follow-up period. For determination of hemoglobin concentration a capillary blood was taken and read by portable spectrophotometer (HemoCue Hb 301 System, Sweden). Hemoglobin values were used to label a subject as having anemia and also categorize it (as mild, moderate, or severe) [40].

### Treatment outcomes and statistical analysis

The study end points and treatment outcomes were defined based on the WHO, 2009 [31] criteria as follows:

#### Primary Outcomes

Early treatment failure (ETF): development of danger signs for severe malaria on days 1, 2 or 3 in the presence of parasitemia; parasitemia on day 2 higher than day 0 count irrespective of axillary temperature; parasitemia on day 3 with axillary temperature ≥ 37.5°C; parasitemia on day 3 ≥ 25% of count on day 0.

Late clinical failure (LCF): development of danger signs for severe malaria after day 3 in the presence of parasitemia, without previously meeting any of the criteria of ETF; presence of parasitemia and axillary temperature ≥37.5°C or history of fever on any day from day 4 to day 28, without previously meeting any of the criteria of ETF.

Late Parasitological Failure (LPF): presence of parasitemia on any day from day 7 to day 28 and axillary temperature <37.5°C, without previously meeting any of the criteria of early treatment failure or late clinical failure.

Adequate Clinical and Parasitological Response (ACPR): absence of parasitemia on day 28 irrespective of axillary temperature without previously meeting any of the criteria of ETF, LTF or LPF.

#### Secondary Outcomes

Fever clearance rate: is proportion of patients whose fever cleared on days 1, 2, and 3.

Parasite clearance rate: the speed at which parasites disappear from the body after drug intake or proportion of patients with negative blood smears on days 1, 2, and 3.

Gametocyte carriage: Proportion of patients with gametocytes during the course of the study.

Time to parasite, fever or gametocyte clearance: is mean time in days (hours) it takes parasite, fever or gametocyte respectively. Data of patients having mixed infection with *P*. *falciparum*, lost to follow-up and vomiting were excluded from the analysis according to WHO recommendations. The proportion of early treatment failure (ETF), late clinical failure (LCF), late parasitological failure (LPF), and adequate clinical and parasitological response (ACPR) at day 28 were calculated. The mean difference, in hemoglobin level on days 0 and 28 and quantitative variables were compared using paired *t* test. P value of < 0.05 was considered a significant level.

Approval was obtained from research and ethics committee of Assasa University prior to data collection. The purpose of the study was explained to the patients and or their parents or legal guardians. Informed written consent was obtained from adult patients or caretakers of child patients aged less than 18 years old.

## Results

### Characteristics of enrolled patients

**Fig 2:**
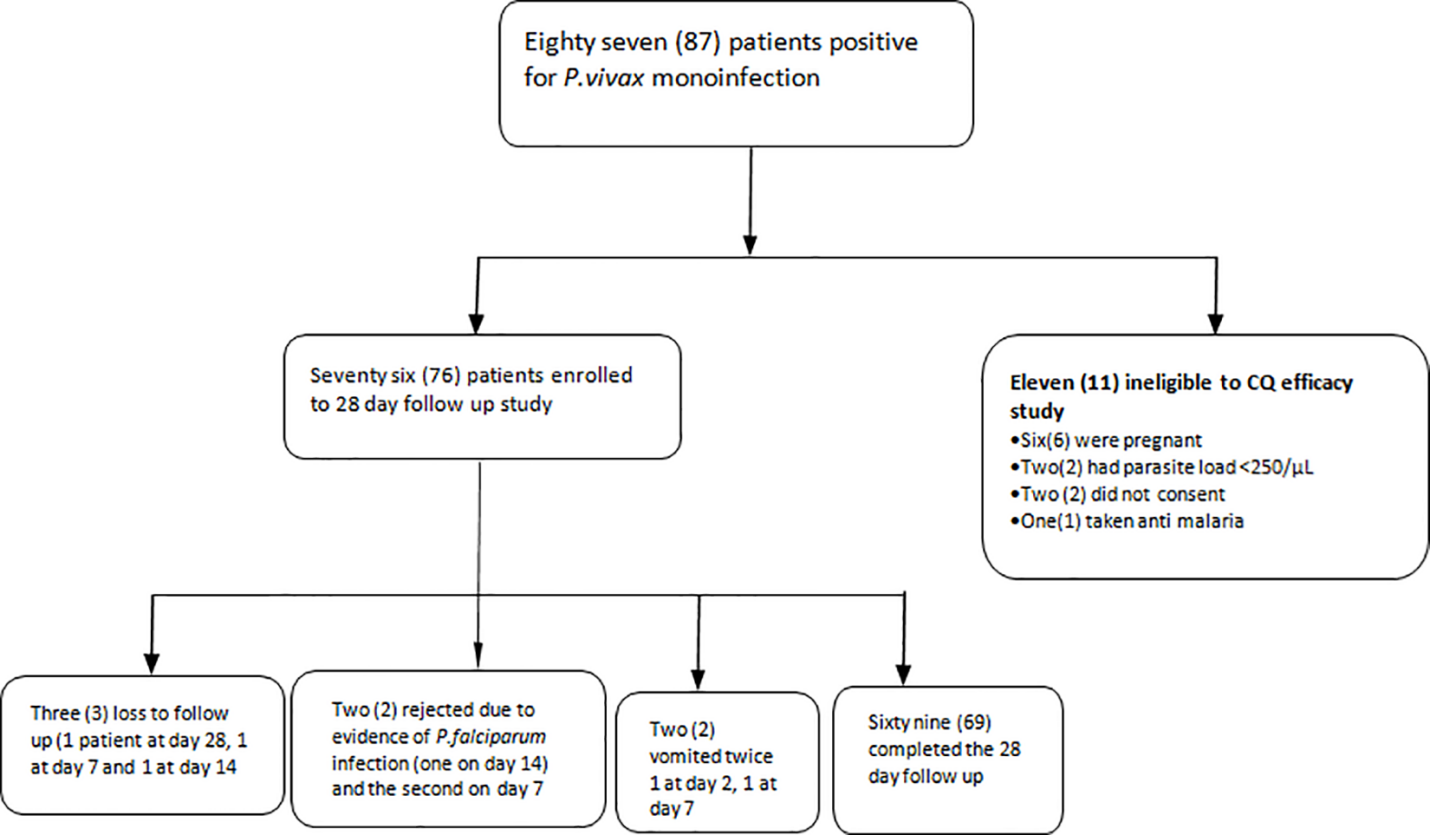
Patient recruitment and follow-up for the trial of CQ efficacy against *P*. *vivax* malaria in Bullen Health Centre Northwest Ethiopia (17 August to 19 December, 2014).

From a total of 1283 clinically suspected individuals who were screened, 209 (16%) were positive for malaria parasites, out of which 87/209 (41.6%) were confirmed to have vivax *malaria* mono-infection. From 87 *P*.*vivax* infected patients, 11 were excluded from being enrolled to this study (6 due to pregnancy, 2 having low parasite density, 2 denial of consent and 1 due to prior intake of anti-malarial drug) (Fig 2). Therefore, seventy six (76) patients were initially enrolled to take part in this 28 day CQ efficacy follow-up study. From these recruited patients 3 (4%), were excluded due to loss to follow and 2 (2.6%) each due to evidence of infection with falciparum malaria, and vomiting during follow up. Finally a total of, 69 patients had fully completed the entire 28 days CQ treatment follow up study and included in the analyses (Fig 2). Initially the study was planned to run from July to November, 2014. However due to logistic and operational reasons it started one month later i.,e on 17 August and ended on 19, December, 2014.

Out of 69 patients enrolled in this study, just over 2/3^rd^ (68%) were males and the remaining 32% were females. The participants age ranged from 3 to 54 years with median (range) of 19 (52). Majority of patients (69.5%) were youngsters (below 25 years old). At the time of follow up, majority (58%) of patients came from outside Bullen town. The mean body temperature at enrollment was 37.9oC (36.2oC - 39Oc) and average body weight was 43 (11–68) kilo grams. At base line, the geometric mean parasitemia was 3540 parasites/micro liters of blood sample in all subjects with average highest parasite load seen in children under the age of 5 years.Over, 80% of patients were febrile (with auxiliary temperature >37oC) at Day 0 (Table 1).

**Table 1:** Baseline characteristics of the study participants, Bullen Health Center, August-December, 2014, Bullen, Ethiopia.

Variable	Age category (in years)			Total (n = 69)
	< 5 (n = 20)	5–14 (n = 15)	≥ 15 (n = 34)	
**Male (%)**	15 (75)	12 (80)	20 (58.8)	47 (68)
**Female (%)**	5 (25)	3 (20)	14 (41.2)	22 (32)
**Median age (Range)**	3.7 (3–4.8)	9.2 (7–13.6)	31(21–54)	19 (3–54)
**Mean weight (Kg)**	17 (11.5–32.7)	27 (15–44.6)	54(27.0–68.0)	43 (11–68)
**Mean body Temperature (**^**o**^**C)**	38.6 (36.4–38.8)	38.1 (36.2–39)	38.0(36.2–39)	37.9 (36.2–39)
**GM parasitemia/μL (Range)**	5211 (2231–9100)	4623(1270–7450)	2320(1880–9201)	3540(1270–9201)
**GM gametocytemia (%)**	17 (85)	13 (86.7)	31 (91)	61/69 (89)
**Mean hemoglobin (g/dl**	14 (10–17.6)	11.2 (7–16.2)	13.2 (7–18)	12.2 (7–15)
**Fever (%)**	19 (95)	11 (73.3)	27 (79.4)	57/69 (82.6)

### Clinical and parasitological study outcomes

Among 57/69 (82.6%) of patients who were febrile at enrolment with auxiliary temperature ≥ 37.5°c, only 4/69 (5.8%) remained so on Day1 and 3/69 (4.3%) on day 2. None of the study patients showed fever on day 3 (Table 2). But one of the patients (1.5%) presented with recurrent fever at day 21 of follow up period.

**Table 2:** Parasite and fever clearance rate and hemoglobin recovery in study participants Bullen Health center, Northwest Ethiopia.

Treatment outcome	Count (%)
**Number analyzed**	69
**Parasite carriage**	
Day 0	69 (100)
Day 1	60 (87)
Day 2	9 (13)
Day 3	2 (3%)
**Fever detected**	
Day 0	57 (82.6)
Day 1	4 (5.8)
Day 2	3 (4.3)
Day 3	0
**Gametocyte carriage**	
Day 0	61(89)
Day 1	26 (37)
Day 2	4 (5.8)
Day 3	0
**TPC[Mean(SD)] in hours**	35 (3)
**TFC[Mean(SD)] in hours**	25 (4.6)
**TGC[Mean(SD)] hours**	28 (3.2)
**Mean(Range) hemoglobin in g/dl**	
Day 0	12.2(7–15)
Day 28	13.3(10–16)

TPC—Time to parasite clearance; TFC- Time to fever clearance; TGC- Time to gametocyte clearance

**Fig 3:**
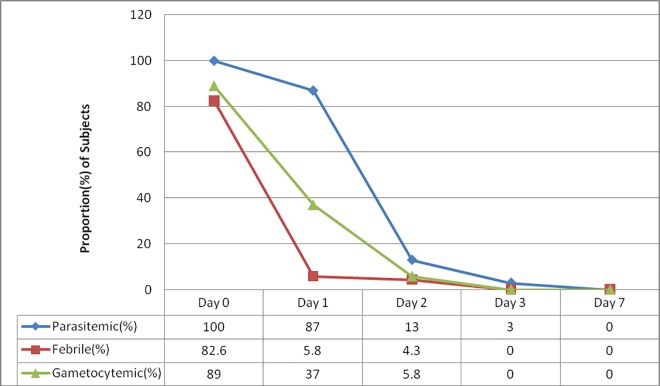
Pattern of parasite, gametocyte and fever clearance in *P*. *vivax* monoinfected patients treated with Chloroquine in Northwest Ethiopia.

As depicted in (Table 1) above, the geometric mean of parasite/μL of blood at enrollment was 3540 with a maximum of 9,201 parasites/μL and minimum of 1270 parasites/μL for asexual parasites and 609/μL for gametocytes. The proportion of parasitemic patients then decreased to 87% (60/69) on day 1, 13% (9/69) on day 2 and 3% on day 3. No parasitemia was seen on day 7 and 21 but one (1.5%) was parasitemic on day 28. Gametocytemia was present in 61/69 (89%) of patients at day 0 and this was dropped to 26 (37%) at day 1 and 4/69 (5.8%) on day 2 and 0% at day 3. No recurrence of gametocytes was reported at any one day of follow up between 7 and 28 (Fig 3).

In this study, parasitemia was observed in 1/69 (1.45%) of afebrile 8 years old girl on day 28 which is classified as Late treatment failure which could be late parasitological failure (LPF). However, there was no gametocyte recovery after day 3. Moreover, one more 6 years old boy showed recurrence of fever on day 21 with parasitemia and was classified as risk of LCF. This gives ACPR rate of 67/69 (97%) or risk of failure in 2/69 (2.9%) of *P*.*vivax* mono-infected patients (Table 3).

**Table 3:** The 28-day cure rate of CQ treatment in *P*.*vivax* mono- infected patients in Northwest Ethiopia (August-December, 2015).

Outcome	Age category(Years)			Total
	< 5	5–14	≥ 15	
	Count (%)	Count (%)	Count (%)	Count (%)
Total analyses	20 (28.9)	15 (21.8)	34 (49.3)	69 (90.8)
ETF	0	0	0	0
LCF	0	1(6.7)	0	1 (1.45)
LPF	0	1(6.7)	0	1 (1.45)
ACPR	20 (100)	13 (86.7)	34(100)	67 (97)
Excluded	3	3	1	7

### Hemoglobin recovery

As shown in Table 4 below, 5/69 (7.4%), 27/69 (39%) and 37/69 (53.6%) of the study participants had an average hemoglobin level between 7–9.9 (moderate anemia), 10–12.9 (mild anemia) and greater than 13g/dL (no anemia) respectively at day 0. However, at the end of follow up, no moderate anemia was found and only 10/69 (14.5%) had mild anemia. Moreover, the proportion of patients presenting with mild anemia dropped from 39% (day 0) to 14.5% (at day 28) (Table 4). None of the subjects had severe anemia (hemoglobin less than 6g/dL) at enrollment or on day 28. The mean hemoglobin level at enrollment was 12.2g/dL (7-15g/dL) and the end of follow up (day 28), it was increased to 13.3g/dl (10-16g/dL). An increase in hemoglobin level between day 0 and day 28 was significant (p = 0.003), and hence likely attributable to chloroquine treatment. However, the change in mean hemoglobin level did not vary among age groups (P = 0.001).

**Table 4:** Hemoglobin concentrations and degree of anemia in *P*. *vivax* infected patients before CQ treatment and at the end of treatment follow up, with respect to age, Northwest Ethiopia (August–December, 2015).

	Age in years (Day 0)								Age in years (Day 28)							
Hemoglobin	1-4(n = 20)		5-14(n = 15)		≥ 15(n = 34)		Total(n = 69)		1-4(n = 20)		5-14(n = 15)		≥ 15(n = 34)		Total(n = 69)	
	n	%	N	%	n	%	n	%	n	%	n	%	n	%	n	%
**< 6**	0	0	0	0	0	0	0	0	0	0	0	0	0	0	0	0
**7–9.9**	0	0	2	40	3	60	5	7.4	0	0	0	0	0	0	0	0
**10–12.9**	7	26	7	26	13	48	27	39	1	10	3	30	6	60	10	**14.5**
**≥ 13**	13	9.7	6	19	18	71	37	**53.6**	19	32	12	20	28	48	59	**85.5**

In this study, some 14/69 (20%) of subjects under follow up developed signs of adverse effects to the treatment of chloroquine (25 mg/kg over 3 days). Among the adverse events, skeletal muscle weakness [6/69 (8.7%)] and abdominal discomfort [4/69 (7.2%)] occurred most frequently. Nausea, dizziness, vomiting and diarrhea was reported in 4/69 (5.8%), 3 (4.3%), 2 (2.9%) and 2 (2.9%) of patients respectively. No adverse effect/s other than those registered in FDA and or national malaria treatments were reported in this study. Adverse effects were shown to occur more frequently among children aged between 1 and 4 years (p = 0.022).

## Discussion

One of the strategies to control malaria is timely provision of effective anti-malarials to infected individuals. Therefore, monitoring the dynamics of anti-malarial drug resistance could help detect emerging resistant strains early. This prospective, observational follow up study assessed the therapeutic efficacy of CQ, which remained a drug of choice for the treatment of *P*.*vivax* mono-infection in Ethiopia, despite the growing concerns of resistance pattern [14,24,34, 41]. In the current study, analysis for CQ efficacy indicators identified a high (97.1%) adequate clinical and parasitological response (ACPR) to the drug. The patient’s hemoglobin level was also improved following parasite and fever clearance e at the end of follow up.

Few studies conducted in Ethiopia so far indicated an alarming levels of CQR [42]. However, most investigators showed that, an adequate clinical and parastological response (ACPR) did not exceed the level (10%) to withdrawal of CQ as first line treatment for *P*.*vivax*. Given, the emergence of chloroquine-resistant vivax malaria can occur at any time in different geographical locations depending on the type of parasite genotypes circulating in the population, it is recommended to conduct similar therapeutic efficacy studies to adequately address the problem of drug resistance.

In this study, the proportion of *P*.*vivax* mono infected subjects were higher compared to other findings in different parts of Ethiopia [27, 43]. Moreover, there was a preponderance of males than that of female participants in current study. Similar findings were reported by other investigators in Ethiopia [41,43,44]. The majority of participants involved in this follow up study were aged below 15 years. This could be attributable to the fact that children in malaria endemic settings show immature immunity compared to adults and hence are likely to develop symptomatic malaria and seek health care services.

Of 69 participants who completed a 28 day follow up in current CQ efficacy study, 2 (2.9%) were labeled as risks of treatment failure and 97.1% ACPR. From the two treatment failures one was a LPF which had occurred on day 28 of follow up period and the remaining one was a LCF that occurred on day 21. Both treatment failures were seen in patients less than 15 years old. Similar scenarios have been reported elsewhere [34,41,45]. Generally, the current ACPR (97.1%) of CQ was implicated as a high efficacy level for treatment of *P*.*vivax* as it is in line with WHO recommendation [9]. A comparable level of cure rate (96.7%) and (98%) was reported in Southern Ethiopia [27]and Debrezeit (Central Ethiopia) [24] respectively.

Similarly a 96.4% cure rate was reported in Serbo (Sothwest Ethiopia) by 2009 [34]. But a study conducted in Halaba in same year [42] showed a significantly reduced cure rate (87%). Moreover, a study from four sites in Southern Ethiopia [41] showed an an overall 9.4% failure rate and 21.9% in one of the study site. A wide range of variability in level of TFs among studies conducted in Ethiopia could be due to the differences in study design, number of participants followed up and geographical as well as *P*.*vivax* genetic polymorphisms.

The current study also showed a fast clearance rate of both parasitemia and fever following CQ mono therapy. Some 98.5% showed no asexual parasitemia on day 3, and only one patient was parasitemic on day 21, and one on day 28 though the density of parasite was minimal. In other studies conducted in Ethiopia [25,34], the rate of parasite clearance was comparable (where by over 80% of subjected cleared parasitemia on day 3). In this study, a significant reduction in gametocyte carriage was one of important finding. Gametocytemia was cleared in all subjects on day 3 and did not reoccurred. However, in a study conducted in Central Ethiopia [25], recurrence of gametocytemia was documented.

In the present study, both mild and moderate types of anemia were relatively prevalent in the pre-treatment phase. However, none of the study subjects with ACPR showed moderate anemia at the end of follow up and a significant reduction in mild anemia was also recorded following therapy. Thus the proportion of patients with anemia (both mild and moderate) was reduced from 46.5% to 14.5% following CQ therapy. Improvement in hemoglobin levels after treatment with anti malarial drugs and successful parasite clearance were reported following treatment of uncomplicated *P*.*falciparum* infections [43,44] with AL and *P*.*vivax* with CQ [27,42]. It is well documented that the hemoglobin concentrations of patients with malaria would be improved at the end of anti-malarial treatment and parasite clearance. Mechanism of anemia is attributable in part to disturbance of erythropoesis by plasmodium induced cytokines and toxins [46,47]. Indeed there are also other causes of anemia such as hook worm infections [48].

In the current study, a three-dose regimen of CQ was safe and well tolerated anti- malarial drug. Some (20%) of subjects showed common adverse effects of CQ and more than one adverse effect per individuals were rarely reported. Weakness and abdominal discomfort were the adverse effects that occurred frequently. The observed common adverse effects occurred more frequently in less than five years old children. The adverse effects that occurred among the study participants were not different from those acknowledged and registered under FDA and national malaria treatment guide of Ethiopia.

This study has got its own strengths and limitations. To the knowledge of authors, it is among few studies of its kind in Ethiopia that would provide useful information on the efficacy of CQ for policy makers. Indeed, abandonment of a given drug is recommended when 10% of infections are not responding to treatment. Moreover, it is relatively cost effectiveness study design particularly in resource constraint settings to monitor the risk of treatment failure regularly. In this follow up study, patients were given directly observed treatment by investigators under supervision and none of them had recurrent vomiting. These conditions are likely to reduce the risk of treatment failure attributable to poor dosing. However, to mention the limitations, we did not determine drug resistance phenotype; neither undertook molecular characterization to determine gene polymorphism, and measured plasma drug concentrations.

## Conclusion

In view of our findings, the risk of treatment failure to three-dose regimen of CQ therapy for vivax malaria is low. A significant improvement in clinical and parasitologic parameters was as well as minimal adverse events were recorded. However, given conflicting reports indicating alarming levels of treatment failures from other sites, there remains a need to monitor the emergence of chloroquine-resistant vivax malaria across the nation to obtain an adequate representation in different ecological and epidemiological settings.

## Supporting Information

S1 File Study protocol. (DOC)

S2 File Questionnaire. (DOC)

S3 File Trend Check list. (DOC)
