# Supplementary material for: Efficacy of Chloroquine for the Treatment of Vivax malaria in Northwest Ethiopia
Source: PLoS One. 2016 Aug 31;11(8):e0161483. doi: 10.1371/journal.pone.0161483 (PMC5007045; doi:10.1371/journal.pone.0161483)
Supplement: S1 File — (DOC) [file pone.0161483.s001.doc]

***ASSOSA UNIVERSITY***

***COLLGE OF HEALTH SCIENCES***

A RESEARCH PROJECT protocol SUBMITTED TO THE OFFICE OF RESEARCH AND COMMUNITY SERVICES OF COLLEGE OF HEALTH SCIENCES, ASSOSA UNIVERSITY.

| **Principal investigator** | Habtamu Bedimo (MSc Medical Parasitology) |
| --- | --- |
| **Co-Investigators** | Yehenew Asmamaw, Melkamu Bedimo, Zelalem Desalegn |
| **Full title of the research project** | Efficacy of Chloroquine for the treatment of *Vivax malaria* in Northwest Ethiopia |
| **Duration of the project** | July to December, 2014 |
| **Purpose of submission** | For evaluation |
| **Study Center** | Bullen Health Center |
| **Total cost of the project** | **46,105.40 birr** |
| **Address of the principal investigator** | Tel: +251-913-221769  Assosa University, Assosa, Ethiopia |

Feb, 2014

Assosa, Ethiopia

**Abstract**

**Background**

Emergence, of resistance to anti-malarials is a major challenge for effective malaria control in sub-Saharan Africa. Extensive use of chloroquine (CQ) as a monotherapy led to significant increase in levels of resistance across many malaria-endemic countries, including Ethiopia. Thus continuous and regular monitoring of drug efficacy is critical for establishing rational anti-malarial drug policies. This study therefore, assesses the therapeutic efficacy of Chloroquine(CQ) for the treatment of *Plasmodium vivax* infections in Northwestern, Ethiopia. **Methods**

An observational, prospective 28-days in vivo therapeutic efficacy evaluation will be conducted from July to December, 2014, in Northwest Ethiopia at Bullen health center. Patients confirmed to have monoinfection of vivax malaria, aged above 6 months will be enrolled. All subjects will be then treated with standard Chloroquine(25 mg/kg for three (3) days). Parasitological and clinical outcomes of treated patients will be evaluated prospectively on days 1, 2, 3, 7, 14, 21, and 28 during the entire follow-up period.. Data will be entered and analyzed using SPSS version 20. Kaplan-Meier survival estimate will be used to analyze therapeutic failure rate during follow-up period. The initial study endpoint will be taken as the risk of failure at 28 days by survival analysis. Changes in fever and parasite clearance will be evaluated at each follow up visit. Changes in mean haemoglobin concentrations will be computed on days 0 and 28 and quantitative variables will be compared using paired *t* test. P value of < 0.05 will be considered a significant level.

**Keywords**

Chloroquine, Efficacy, *vivax malaria*

**Introduction**

There are five species of plasmodia known to infect humans of which, *Plasmodium vivax* is the most widely distributed species responsible for 25-40% of malaria cases worldwide [1], and substantial morbidity associated with vivax malaria has been reported [2-4]. In Ethiopia, vivax malaria accounts for approximately 40% of all malaria cases, and most recently it seems, this proportion is even rising. Despite the public health importance, *P. vivax* malaria has received little attention and limited funds for research and control, since it usually produces less severe symptoms than falciparum malaria [2, 5, 6].

The current treatment for vivax malaria relies primarily upon two anti-malarial drugs, chloroquine (CQ) and primaquine (PQ), with the latter being the only effective drug against the hypnozoite stage. In areas where susceptibility to CQ is ascertained the recommended doses are CQ 25 mg/kg bw (body weight) during three consecutive days (10, 10 and 5 mg/kg bw) and Premaquine(PQ) at 0.25–0.75 mg/kg bw during 14 days [7,8]. Of course, the 14-day treatment effectively cures primary blood infections and is the most effective for preventing relapses [9]. However, in areas where CQR is substantial, it is recommended that artemisinin combination therapy (ACT) be used instead, also in combination with PQ for radical cure [8].

The first evidence of CQ resistant (CQR) to *P. vivax* began to emerge in 1989 [10, 11], 30 years after the documentation of CQR *Plasmodium falciparum*. Since, then the problem of CQR to *plasmodium vivax* continued to occur in different regions at varying degrees. For instance, in Indonesia, East Timor and Papua New Guinea, CQ-resistant vivax malaria has already reached an alarming prevalence [12]. CQR has also occurred in Latin American countries such as (Guyana, Peru and Brazil) [13]. The four clinical trials carried out in Asia (Thailand and Pakistan) and Africa (Ethiopia), for instance, showed that CQ alone (25 mg/kg over 3 days) is less effective against *P. vivax* asexual blood stages than CQ (25 mg/kg over 3 days) co-administered with PQ (15 mg of PQ base/day for 14 days) over 28 days of follow-up [14].

The emergence of resistance to anti malarials threatens efforts towards malaria control and elimination. Resistance to Drugs has been implicated in the spread of malaria to new areas and re-emergence of malaria in areas where the disease had been eliminated. It also plays significant role in the occurrence and severity of epidemics in some parts of the world [15] and results in an ongoing diseases transmission. Therefore, there is a need to monitor, the status of drug resistance to anti-malarials on continuous and regular basis (often at 2 years interval. To do so, three main tools are used for drug resistance monitoring and assessing which includes therapeutic efficacy tests (*In* vivo*)*, in vitro tests, and analyses of molecular markers.

Therapeutic efficacy assessment according to the standard protocol of the WHO is the most useful for updating national treatment policies. These include, therapeutic assessment based on evaluation of the clinical and parasitological outcomes remains the mainstay of monitoring the efficacies of anti-malarial regimens and is recommended [16]. Measures such as parasite clearance time, fever clearance time or gametocyte clearance time in vivo and in vitro assays are used to indirectly detect any variation in parasite sensitivity thereby facilitating early warning in case of emergence of tolerance or resistance [17, 18].

In Ethiopia, Chloroquine 150 mg base tablet *OR* chloroquine syrup 50 mg base is the first line drug of choice for the treatment of vivax malaria. The ideal dose is 10 mg base/kg po immediately (Day 1), followed by 10 mg base/kg po at 24 hours (Day 2), and 5mg base/kg po at 48 hours (Day 3) for a total dose of 25 mg chloroquine base/kg over three days with a maximum total of 1,500 mg chloroquine base (maximum of 2,500 mg chloroquine phosphate salt) over three days in three divided doses [19].

Although CQ has been the cheapest and mainstay, treatment for vivax, malaria in Ethiopia, its efficacy is not well established, throughout the country. There is, little information on the response of *P. vivax* to CQ in Ethiopia. To mention them, a study conducted from Debre Zeit area in 1996, the rate of parasitological failure to CQ treatment on day 7was indicated to be 2% (n = 255)[20] and more recently an increased overall failure rate of 5.76% at same area[21]. Such growing evidences of the occurrence of CQR at varying degrees in Ethiopia necessitate further regular assessment of the drug’s treatment outcome in different setting to help support policy makers if changes in treatment policies are to be made. Therefore, in the present study, we planned to assess the efficacy of standard CQ treatment for *P. vivax* malaria in a one arm observational, prospective follow up study among patients who will be visiting the outpatient department of Bullen Health Center, Northern, Ethiopia.

**Significance of the study**

Antimalarial Drug resistance is a problem that contributes an estimated 90% of the malaria burden out of the total cases. So, in vivo test of CQ efficacy in Metekel, Bullen district, one of malaria endemic areas in Ethiopia, based on WHO protocol, will be benchmark for further study and it gives a clue on the current situation of CQ affectivity. Therefore conducting a study in this area brings the problem in to light; is one of the means to formulate recommendations and to assist the regional ministry of health making informed decisions on current national antimalar­ial treatment guidelines, particularly of vivax malaria.

**Methods**

**Study area**

The BFGS has an estimated area of 51,000 square kilometers and shares common borders with the State of Amhara in the east, the Sudan in the north-east, and the State of Oromia in the south. It is divided into 3 administrative zones, 19 Weredas and 33 Kebeles (the smallest administrative units) [22].  Metekel is the largest zone with an area of 26,272 square kilometers followed by Assosa and Kamashi. The state has diverse topography and climate. The later includes the familiar traditional zones - "kola", "dega", and "woyna dega". "About 75% of the State is classified as "kola" (law lands) which is below 1500 meters above sea level. The altitude ranges from 550 to 2,500 meters above sea level. The average annual temperature reaches from 20-25^0C^. During the hottest months (January - May) it reaches a 28 - 340C. Bullen is one of the town in the Metekel zone of BGRS which lies on a longitude of 10°0^o^0°N 39°590 E36°0'0"E and latitude of 12°0'0"N. The town has a total of 30,828 inhabitants, and malaria is implicated to be one of the major health problem in the area [23].

**Fig 1**. Map of study site

**Population**

**Source population**

Patients who are ≥6 months old and visiting Bullen health center from July to December, 2014 in Bullen district will be considered as the source population.

**Study population**

Patients attending the health center with symptoms suggestive of malaria, are the study population.

**Study subject**

People who are *plasmodium vivax* mono infected and ≥6 months old and fulfills the inclusion criteria of WHO will be enrolled for quantitative study. All adult patients will sign an informed consent form for participation. Parents or guardians will give informed consent on behalf of children in age of less than 18 years.

**Inclusion and exclusion criteria**

Patients will be selected on the basis of the following WHO criteria:

- Patients ≥6 months and above
- Absence of severe malnutrition characterized by standard of WHO protocol
- A slide-confirmed infection with *P.vivax* only (i.e. no mixed infections).
- Initial parasite densities greater than 250 asexual parasites/μl
- Patients with no other signs of severe and complicated malaria according to current WHO definitions
- Having the measured axillary temperature ≥37.5 °C
- Ability to attend the stipulated follow-up visits, and easy access to the health facility.
- Informed consent provided by patient or parent/guardian
- ability to swallow oral medication;
- absence of concomitant febrile diseases
- absences of history of allergic reaction to CQ treatment
- ability and willingness to comply with the protocol for the duration of the study and to comply with the study visit schedule;

Individuals who did not fulfill the above criteria and the followings will not include:

- Study subjects who are Pregnant or lactating or
- The existences of underlying chronic severe illness (e.g. cardiac, renal, hepatic diseases, HIV/AIDS) will not consider as study subjects.

**Loss to follow-up**

Loss to follow-up will be deemed to occurs when, despite all reasonable efforts, an enrolled patient does not attend the scheduled visits and cannot be found. No treat­ment outcome will be assigned to these patients. Every effort will made to schedule a follow-up visit for patients who fail to return to the study site, especially during but also after administration of the drug. These patients will be classified as lost to follow-up and censored or excluded from the analysis. Patients who are lost to follow-up but who subsequently return to the study site before day 28 will not be turned away and will be encouraged to return for check-up visits.

**Protocol violation and withdrawn**

Study patients who meet any of the following criteria will be classified as withdrawn.

- Withdrawal of consent, a patient may withdraw consent at any time, without prejudice for further follow-up or treatment at the study site.
- A patient who vomits the study medication twice will be withdrawn from the study and given rescue treatment.
- failure to attend the scheduled visits during the first 3 days
- Serious adverse events necessitating termination of treatment before the full course is completed.
- Severe malaria on day 0
- if self-or third-party administration of anti­malarial drug (or antibiotics with antimalarial activity)
- Occurrence during follow-up of concomitant disease that would inter­fere with a clear classification of the treatment outcome;
- Detection of a mono-infection with another malaria species during follow-up
- Misclassification of a patient due to a laboratory error (parasitaemia), leading to administration of rescue treatment

**Hypotheses**

The efficacy of Chloroquine for the treatment of uncomplicated vivax malaria remained high in Northern Ethiopia

**Objectives**

**General objective:**

- To assess the therapeutic efficacy and safety of CQ monotherapy for the treatment of uncomplicated *vivax malaria* in Bullen district, west Ethiopia.

**Specific objectives**

- To assess the clinical efficacy of CQ among ≥ 6 months old who suffer from uncomplicated vivax malaria
- To measure the parasitological efficacy of CQ among ≥6 months who suffer from uncomplicated vivax malaria; and
- To assess the incidence of adverse events in vivax malaria mono infected patients who take CQ treatment

**Sample size**

The sample size will be determined using a simple population proportion formula by taking the proportion of treatment failure of the town, which is unknown. Therefore, according to WHO protocol, the value of population proportion will be 0.50, which helps to have the largest sample size for enough observations, irrespective of the actual failure rate value of the true population. So according to population proportion formula, the expected population proportion of clinical treatment failures (*P=0.5*) in the confidence interval (usually 95%), and the precision (*d*) usually 10%, this study will have 422 subjects including 10% of non-respondents.

n= (z^2^_α/2_) pq

d^2^

n = (1.96)^2^(0.5x0.5) = 384,

(0.5)^2^

Therefore, 422 subjects will be included in this study to have high amenable observations. However, as WHO in 2009 suggests that, to say anti-malaria therapeutic test result is representative in a given area when at least 50 and above sample size will be considered.

**Measurements**

**Variables**

**Dependant variables**

- A 28 days of CQ efficacy/clinical and parasitological outcome after treatment through the following methods:
- **Hemoglobin concentration:** Hemoglobin will be determined through Hem cue heamatological analyzer or through haematocrit at days of 0, and 28 of time schedule to check the improvement of the patients’ heamoglobine level, which indicates indirect evaluation of the drug efficacy.
- **Clinical signs and symptom consideration:** the clinical outcome of patients after treatment will be assessed and recorded according to the protocol of WHO (2009).
- **Parasitic loads:** Parasitic load will be quantitate according to the schedule of WHO protocol (day 0, 1, 2, 3, 7, 14, 21and 28 day), and will help for the classification of the drug response.
- Early Treatment Failure (ETF)
- Late Clinical Failure (LCF)
- Late Parasitogical Failure (LPF)
- Adequate Clinical and Parasitological Response (ACPR)
- **Time to fever clearance (FCT)**: Time to fever clearance will be record according to the WHO protocol.
- **Safety and tolerability**: The incidence of any adverse event will be documented. All patients will be asked routinely about previous symptoms and about symptoms that have emerged since the previous follow-up visit through checklists. When clinically indicated, patients will be evaluated and treated appropriately. All adverse events will be recorded on the case report form.

**Independent variables**

- **Socio-demographic variables:** Questionnaire will be developed which can assess sex, age, and educational status with socio-economic status of the population.
- **Body weight:** Body weight will be recorded on day 0 to the nearest kilogram on a Salter scale or on a hanging scale for young children.

**Data /sample collection**

Capillary Blood collection will done by the senior laboratory technicians who have long time experience, for screening (whether the subjects are *p.vivax* positive or not) and if the sample is positive, the person will be asked for his/her consent to participate in the study up to end. After having the consent blood will be run for hematological procedure (heamoglobine determination) for 0, and 28 days and for thick and thin blood smear preparation according the schedule of the study.

**Quality control**

Clinical examination and parasite counts will be perform every day for two consecutive days whether the parasites and symptoms are completely cleared or not and continuous check up will be perform weekly from day 7 of the first drug administration till 28 days of total follow up days. Axillary temperatures will record every day of the first two days during blood sample collection. Primary end points of the study are the parasitological and clinical response to treatment. All slides on day 0, all positive slides after day 7 (regardless of the parasite species) and 10% randomly selected negative slides will crosschecked by a senior laboratory technician who are engaged in the regional laboratory. The machine that can measure heamoglobin will be calibrated and adjust through standards and, reagents like Giemsa stains will be check by the known positive samples. In addition, for the laboratory technicians, if necessary refreshment course on the identification and enumeration of the parasites will be provide.

**Data analysis**

Data will check for its completeness before entering for analysis. Then it will be entered after edited and cleaned in to SPSS 20.0 version for analysis. The overall CQ efficacy will be determined and recorded through the help of descriptive and inferential method of data analysis. Simple frequencies will be applied to see the distribution of the socio demographic characteristics of independent variables. Kaplan-Meier survival estimate will be used to estimate therapeutic failure risk during follow-up period. The mean difference, in haemoglobin/hematocrit level on days 0 and 28 and quantitative variables will be compared using paired *t* test. P-value less than 0.05 will take as statistical significance association and 95% CI of Odds ratio for the strength of the statistical associations. Then it will code and transcribed manually from the notes and recorded and will supplement to the results of the qualitative study accordingly.

**Ethical consideration**

This protocol will be submited to the collage of Public Health and Medical Sciences of ethical committee of Assasa University for approval and obtain ethical clearance letter from the committee before data collection start. A written letter of permission from BGRS Health Bureau and Drug Administration and Control Agency (DACA) will be obtained prior data collection. Written and oral consent of each respondent will also obtain to participate on the study after explaining the aim of the study.

**Limitation and strength of the study**

**Limitation of the study**

Even though it is seasonal, Bullen district is one of the malarious areas in Ethiopia, which has high malaria transmission rate, and so within the follow up of 28 days of WHO protocol, there may be a possibility of re-infection, which cannot be identified without the help of molecular technique (PCR).

**Strength of the study**

The study will use WHO protocol for the efficacy test of CQ. In addition, it provides information on the drug effectiveness status of the town and gives strength for interpretation of the drug response in combination with other parameters.

**Operational definitions and study end points/outcomes**

**Early treatment failure (ETF):** includes any of the following:

• Development of danger signs or severe malaria on Day 1, Day 2 or Day 3, in the presence of parasitaemia

• Parasitaemia on Day 2 higher than Day 0 count irrespective of axillary temperature

• Parasitaemia on Day 3 with axillary temperature ≥37.5 °C

• Parasitaemia on Day 3 ≥ 25% of count on Day 0.

**Late Clinical Failure (LCF)**

• Development of danger signs or severe malaria after Day 3 in the presence of parasitaemia, without previously meeting any of the criteria of *Early Treatment Failure*

• Presence of parasitaemia and axillary temperature ≥ 37.5 °C (or history of fever) on any day from Day 4 to Day 28, without previously meeting any of the criteria of *Early Treatment Failure*

**Late Parasitological Failure (LPF)**

• Presence of parasitaemia on any day from Day 7 to Day 28 and axillary temperature < 37.5 °C, without previously meeting any of the criteria of *Early Treatment Failure* or

*Late Clinical Failure*

**Adequate Clinical and Parasitological Response (ACPR)**

Absence of parasitaemia on Day 28 irrespective of axillary temperature without previously meeting any of the criteria of Early Treatment Failure or Late Clinical or Failure or Late Parasitological Failure is taking as ACPR.

**Time to fever clearance (FCT)**: Time to fever clearance is define as time from first dose until the first time the body temperature decreased below and remained below 37.5° C for at least a further 48 hours.

**Safety and tolerability -** The incidence of any adverse event will be documented. All patients will be asked routinely about previous symptoms and about symptoms that have emerged since the previous follow-up visit. When clinically indicated, patients will be evaluated and treated appropriately.

**Parasite clearance time**- defined as the first thick film negative for two consecutive days and

**Fever clearance time** - axillary temperature below 37.5^0^C for two consecutive days

**Sever malaria:** Severe manifestation of malaria in adults and children will be considered through Clinical manifestations and Laboratory findings.When a patient manifest Prostration, impaired consciousness, respiratory distress, multiple convulsions, circulatory collapse, pulmonary edema, abnormal bleeding, jaundice and haemoglobinurea clinically moreover, laboratory results helps to confirm the disease severity. Findings which help for classification of Severe malaria includes sever anaemia having haemoglobin level of < 5 g/dl or haematocrit < 15%. In addition, hypoglycemia with blood glucose level < 2.2 mmol/l or 40 mg/dl, acidosis (plasma bicarbonate < 15 mmol/l), hyperlactataemia (venous lactic acid > 5 mmol/l), hyperparasitaemia (>4% in non-immune patients), renal impairment (serum creatinine above normal range for age) and among children the occurrence of two or more convulsions within 24 h and persistent vomiting will be considered as sever malaria infection.

**Uncomplicated malaria:**

**Resistance**: the ability of a parasite strain to survive and/ or multiply despite the administration and absorption of a drug given in doses equal to or higher than those usually recommended but within the tolerance of the subject.”

**Dissemination of the result**

The final report will be disseminate to the collage of public health and medical sciences of Assosa University, BGRS health bureau and an effort will be made to present to the scientific community through publications and presentation; to the broader community through magazine and other channels in and outside.

**Work plan(2014)**

| Tasks to be Performed | Responsible Person | periods | | | | | | | | | | | |
| --- | --- | --- | --- | --- | --- | --- | --- | --- | --- | --- | --- | --- | --- |
|  |  | May | Jun. | Jul. | Aug. | Sep. | Oct. | Nov. | Dec. | Jan. | Feb. | Mar. | Apr. |
| Topic selection | PI |  |  |  |  |  |  |  |  |  |  |  |  |
| Approval & Finalize research proposal | PI |  |  |  |  |  |  |  |  |  |  |  |  |
| Submission of it to ethical clearance committee | PI |  |  |  |  |  |  |  |  |  |  |  |  |
| Collection of budget, material and other resources | PI |  |  |  |  |  |  |  |  |  |  |  |  |
| Written consent from respective bodies | PI |  |  |  |  |  |  |  |  |  |  |  |  |
| Training data collectors | PI |  |  |  |  |  |  |  |  |  |  |  |  |
| Data collection and organization | PI |  |  |  |  |  |  |  |  |  |  |  |  |
| Data analysis & interpretation and draft write up | PI |  |  |  |  |  |  |  |  |  |  |  |  |
| Final report dissemination | PI |  |  |  |  |  |  |  |  |  |  |  |  |
| Monitoring and evaluation | PI |  |  |  |  |  |  |  |  |  |  |  |  |

# Budget break down

Chemicals and reagents

| Serial no | Items | Unit | Quantity | Unit Price | | Total Price | | |
| --- | --- | --- | --- | --- | --- | --- | --- | --- |
|  |  |  |  | Birr | Cents | Birr | | Cents |
|  | Microscopic slide | Pack | 20 | 49 | 00 | 980 | 00 | |
|  | RDT | Kits | 550 | 15 | 00 | 8250 | 00 | |
|  | Cotton | pack | 5 | 14 | 75 | 82 | 35 | |
|  | 70% Alcohol | Liter | 5 | 18 | 20 | 91 | 00 | |
|  | Syringes | Pack | 450 | 10 | 00 | 4500 | 00 | |
|  | EDTA Test tube | Pack | 100 | 25 | 00 | 2500 | 00 | |
|  | Lancet | Pack | 3 | 40 | 35 | 121 | 15 | |
|  | Giemsa stains | Liter | 15 | 20 | 00 | 300 | 00 | |
|  | Methyl alcohol | Liter | 2 | 90 | 85 | 181 | 70 | |
|  | Distilled water | Liter | 20 | 10 | 25 | 205 | 00 | |
|  | Oil immersion | Bottle | 3 | 10 | 45 | 31 | 15 | |
|  | 5% Bleach | Liter | 5 | 100 | 00 | 500 | 00 | |
|  | Washing soap | Pcs | 10 | 10 | 00 | 100 | 00 | |
|  | Slide box | Pieces | 4 | 45 | 75 | 182 | 15 | |
|  | Digital camera | Pieces | 1 | 2000 | 00 | 2000 | 00 | |
|  | Examination Glove | Pack | 5 | 30 | 00 | 150 | 00 | |
|  | Heavy duty glove | Pcs | 5 | 20 | 00 | 100 | 00 | |
|  | Capillary tubes | Pack | 5 | 100 | 00 | 500 | 00 | |
|  | Sealant | Pack | 3 | 50 | 00 | 150 | 00 | |
| **Sub total** | | | | | | **20, 923** | **00** | |

**Stationary materials**

| S.No. | Items  Materials | Unit | Quantity | Unit price | | Total price | |
| --- | --- | --- | --- | --- | --- | --- | --- |
|  |  |  |  | Birr | Cent | Birr | Cent |
|  | Typing paper | Pieces | 3 | 80 | 00 | 240 | 00 |
|  | Duplicating paper | Pieces | 20 | 75 | 00 | 1500 | 00 |
|  | Pen | Pcs | 7 | 2 | 00 | 14 | 00 |
|  | Pencil | Pcs | 15 | 0 | 50 | 7 | 50 |
|  | Binder | Pieces | 10 | 5 | 00 | 50 | 00 |
|  | Flash disc | Pieces | 1 | 250 | 00 | 250 | 00 |
|  | CD | Pieces | 5 | 10 | 00 | 50 | 00 |
|  | Fuel | Litter | 50 | 12 | 00 | 600 | 00 |
|  | Marker | Pcs | 10 | 20 | 00 | 200 | 00 |
| Total | | | | | | **2911** | **00** |

Man power

| No | Man power | Quantity | Fee/day | Total working days | Total fee in birr |
| --- | --- | --- | --- | --- | --- |
|  | Physician | 1 | 140 | 25 | 3500 |
|  | Data collector (Nurses) | 2 | 70 | 25 | 3500 |
|  | Laboratory Technologists | 4 | 70 | 25 | 7000 |
|  | Driver | 1 | 58 | 20 | 1160 |
|  | Supervisor | 1 | 70 | 20 | 1400 |
|  | Technical staff | 1 | 70 | 15 | 1050 |
|  | Secretary | 1 | 47 | 10 | 470.00 |
| Sub Total | | | | | **18, 080** |

Grand total = 20923+2911+18080 = **41914**

10 % contingency from materials = **4191.4** birr

Therefore, net budget = **41914+4191.4** = **46,105.40 birr**

**References**

1. Gething PW, Elyazar IR, Moyes CL, Smith DL, Battle KE, Guerra CA, et al. A long neglected world malaria map: Plasmodium vivax endemicity in 2010.PLoS Negl Trop Dis. 2012; 6:e1814.
2. Anstey NM, Russell B, Yeo TW, Price RN. The pathophysiology of vivax malaria. Trends Parasitol. 2009; 25:220–7.
3. Tjitra E, Anstey NM, Sugiarto P, Warikar N, Kenangalem E, Karyana M, et al. Multidrug-resistant Plasmodium vivax associated with severe and fatal malaria: a prospective study in Papua. Indonesia PLoS Med. 2008;5:e128.
4. Genton B, D’Acremont V, Rare L, Baea K, Reeder JC, Alpers MP, et al. Plasmodium vivax and mixed infections are associated with severe malaria in children: a prospective cohort study from Papua New Guinea. PLoS Med. 2008;5:e127.
5. Mendis K, Sina BJ, Marchesini P, Carter R. The neglected burden of Plasmodium vivax malaria. Am J Trop Med Hyg. 2001;64:97–106.
6. Mueller I, Galinski MR, Baird JK, Carlton JM, Kochar DK, Alonso PL, et al. Key gaps in the knowledge of Plasmodium vivax, a neglected human malaria parasite. Lancet Infect Dis. 2009;9:555–66.
7. White NJ. Drug resistance in malaria. Br Med Bull. 1998;54:703–15.
8. WHO. Guidelines for the treatment of malaria. World Health Organization, Geneva. 2015. <http://www.who.int/malaria/publications/> atoz/9789241549127/en/. Accessed 30 Sep 2015.
9. Galappaththy GN, Omari AA, Tharyan P. Primaquine for preventing relapses in people with *Plasmodium vivax* malaria. Cochrane Database Syst Rev. 2007; 1:CD004389.
10. Rieckmann KH, et al. *Plasmodium vivax* resistance to chloroquine? Lancet. 1989; 2:1183–1184.
11. Baird JK, et al. Resistance to chloroquine by *Plasmodium vivax* in Irian Jaya, Indonesia. Am. J. Trop. Med. Hyg. 1991; 44:547–552.
12. Baird JK. Resistance to therapies for infection by Plasmodium vivax. Clin Microbiol Rev. 2009;22:508–34.
13. Vaca G, Arámbula E, Esparza A. Molecular heterogeneity of glucose- 6-phosphate dehydrogenase deficiency in Mexico: overall results of a 7-year project. Blood Cells Mol Dis. 2002;28:436–44.
14. Galappaththy GN, Omari AA, Tharyan P. Primaquine for preventing relapses in people with *Plasmodium vivax* malaria. Cochrane Database Syst Rev. 2007;1:CD004389
15. Foster SD. Pricing, distribution, and use of antimalarial drugs. *Bulletin of the World Health Organization* 1991;69:349–363.
16. WHO (2014) World Malaria Report 2014. World Health Organization,Geneva
17. Sowunmi A, Adewoye EO, Gbotsho GO, Happi CT, Sijuade A, Folarin OA, et al. Factors contributing to delay in parasite clearance in uncomplicated falciparum malaria in children. Malar J. 2010;9:53.
18. Stepniewska K, Ashley E, Lee SJ, Anstey N, Barnes KI, Binh TQ, et al. In vivo parasitological measures of artemisinin susceptibility. J Infect Dis. 2010;201:570–9.
19. Federal ministry of Health(FMoH). **NATIONAL MALARIA GUIDELINES Third Edition** . **Addis Ababa January 2012.**
20. Tulu, N.A., Webber, R.H., Schellenberg, A., Bradley, D.J., 1996. Failure of chloroquine treatment for malaria in the highlands of Ethiopia. Trans. R. Soc. Trop. Med. Hyg.90, 556–557.
21. Asnakew K. Yeshiwondim, Afework H. Tekle, Dereje O. Dengela, Ambachew M. Yohannes, Awash Teklehaimanot. Therapeutic efficacy of chloroquine and chloroquine plus primaquine for the treatment of Plasmodium vivax in Ethiopia. Acta Tropica 113 (2010) 105–113
22. <http://www.ethiopar.net/>
23. <http://www.ethiodemographyandhealth.org/Benishangul.html>

**Annex-1 Laboratory procedures**

**3.1.** Method of sample collection capillary blood collection

1. Rub the site vigorously with cotton or a gauze pad moistened with 70% alcohol
2. After the skin has dried, make a puncture 2-3mm deep with a sterile lancet. A rapid and firm puncture should be made with control of the depth
3. a freely flowing blood should be taken and will make thick and thin blood films.
4. Stop the blood flow by applying slight pressure at the site preferably with a gauze pad

Preparation of thick blood film

- Place a small drop of blood on a clean slide
- spread it with an applicator stick or the corner of another slide until small prints are just visible through the blood smear
- The prepared smear corresponds to a circle of approximately 2cm diameter.

Preparation Thin blood film

1. Place a small drop of well mixed EDTA blood (about 2-3 mm) or freshly collected from capillary blood sites , 1.0 cm from the end of the glass slide, using either a plain capillary tube or other type of blood dropping device

- when the blood is from an anemic patient larger drop of blood should be used
- If capillary blood to be used, the procedure will follow SOP for proper collection of capillary blood

1. the spreading slide is placed in front of the drop of blood at an angle of about 30^o^ - 40 ^o^ to the slide and then is moved back to make contact with the drop (both the spreader and the base slide should be free from grease)
2. The drop will spread out quickly along the line of contact of the spreader with the slide
3. As the drop of blood spreads, be careful not to let it spread to both edges of the spreader
4. The spreader is advanced with a smooth steady motion so that a thin film of blood is spread over the slide
5. Allow the smear to air-dry

- Also do not use heat for drying

1. Label the name of the patient and date or reference number is written on the head of the film using a lead pencil, a diamond marker or a graphite

3.2. Giemsa stain

- Place the slides in a staining rod/rack
- Do not fix the films before staining.
- Cover the air-dried smear with a 1:10 diluted Giemsa using buffered distilled water at pH 7.2 (recommended for malaria parasites in order to stain schuffner’s granules) as a diluents
  - 1:10 Giemsa =1 part of stock Giemsa + 9 parts buffered water
- stain the slides as follows:
  - 30 min if using 3% stain solution
  - 10 min if using 10% stain solution
- Wash the stain from the slide gently using clean water (not necessarily distilled water or buffered water)

Wipe the back of each slide clean and place it in draining rack for the preparation to air dry

3.3. Heamatocrite procedure

- If capillary blood from finger puncture is to be used, follow SOP for capillary blood collection
- If anticoagulated venous blood, mix specimen well
- Allow the blood to enter the tube by capillarity
- Fill 3/4^th^ of the capillary tube ; do in duplicate
- Seal the capillary tubes by vertically placing the dry end into a tray of sealing compound (wax or plasticin)
- Rotate the capillary tube slightly and remove it from the tray. The sealant plug should be 4-6mm long. Inspect the seal for a flat bottom.

Place the filled, sealed capillary tube in the groove (slots) of the centrifuge with the sealed end toward the periphery.

- Set the timer of the centrifuge at 5 minute and spin at 10,000-15,000g.
- Read the PCV using a reading device that is either part of the centrifuge or separate from it

**Microscopic blood examination**

Thick and thin blood films for parasite counts will be obtained and exam­ined at screening on day 0 to confirm adherence to the inclusion and exclu­sion criteria. Thick blood films will be also examined on days 2, 3, 7, 14, 21and 28, or any other day if the patient returns spontaneously and parasitological reassessment is required. Specimens will be labelled anony­mously (screening number or study number, day of follow-up, date).

A fresh Giemsa stain dilution (10%v/v) will be prepared at least once a day and possibly more often, depending on the number of slides to be processed. Giemsa-stained thick and thin blood films will be examined at a magnifica­tion of 1000× to identify the parasite species and to determine the parasite density.

Two blood slides per patient will be obtained: one thick blood smear and one thin blood smear. Thick blood slide will be stained by 10% Giemsa for 10–15 min for initial screening. Moreover, if the patients are subsequently enrolled, the same staining will be used for all slides obtained at follow-up visits. The study number of the patient, the date and the day of follow-up will be recorded either on the frosted edge of the slide or on the glass with a permanent glass pen.

The thick blood smear for initial screening will be used to count the numbers of asexual parasites and white blood cells in a limited number of microscopic fields.

Thin blood smear will be used to calculate the parasite density, by counting the number of asexual parasites in a set number of white blood cells (typically 200), using the following formula:

Parasite density (per µl) =
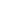

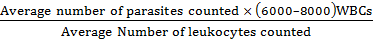


The same technique will be used to establish the parasite count on each subsequent blood film. When the number of asexual parasites is less than 10 per 200 white blood cells in follow-up smears, counting will be done against at least 500 white blood cells (i.e. to completion of the field in which the 500^th^ white blood cell is counted). A blood slide will be considered negative when examination of 1000 white blood cells reveals no asexual parasites. The presence of gametocytes on an enrolment or follow-up slide will be noted, but this information will not contribute to basic evaluation.

Two qualified microscopists will read all the slides independently, and para­site densities will be calculated by averaging the two counts. Blood smears with discordant results (differences between the two microscopists in species diagnosis, in parasite density of > 50% or in the presence of parasites) will be re-examined by a third, independent microscopist, and parasite density will be calculated by averaging the two closest counts.

Annex-: Dosing chart of CQ

Tablets of Chloroquine 150mg base or syrup 50mg base per 5ml(note: one 250mg chloroquine phosphate salt tablet contains 150mg chloroquine base). Total dose of 25 mg base per kg over 3 days(10mg base per kg on day 1, 10 mg base per kg on day 2 and 5 mg base per kg on day 3).

| **Weight(Kg)** | **Age** | **Dosage** | | |
| --- | --- | --- | --- | --- |
|  |  | **Day 1** | **Day 2** | **Day 3** |
| 7-10 | 4-11 months | ½ tablet | ½ tablet | ½ tablet |
| 11-14 | 1-2 years | 1 tablet | 0.5 tablet | 0.5 tablet |
| 15-18 | 3-4 years | 1 tablet | 1 tablet | 1tablet |
| 19-24 | 5-7 years | 1 ½ tab | 1 ½ tab | 1 tab |
| 25-35 | 8-11 | 2 ½ tab | 2 tab | 1 tab |
| 36-50 | 12-14 | 3 tab | 2tab | 2 tab |
| 51 and above | 15 yrs and above | 4 tab | 4 tab | 2 tablets |

**Annex-5: Consent form**

**Assosa University, Ethiopia**,

**Dear Participant**

My name is Habtamu Bedimo, and I am a facult at Addis Ababa University. I am going to conduct a study on the treatment outcome of malaria. Malaria is a dangerous disease; however, it can be treated with medicine. The purpose of this study is to confirm that the medicine, called Chloroquine, is still effec­tive for curing vivax malaria. This study will be approved by the Ethical clearance committee of Assosa University, college of health sciences, ethical committee.

I am inviting all adults and children aged ≥6 months living in Bullen and its surrondings to take part in this study.

Now, I am going to give you information and invite you to participate in this surveillance study. Before you decide whether to participate, you can talk to anyone, you feel comfortable with. There may be some words that you do not understand. Please ask me to stop as I go through the information, and I will take time to explain. If you have questions later, you can ask doctor, staffs or me.

Your participation in this study is entirely voluntary. If you choose not to consent, all the services you receive at this clinic will continue as usual. Even if you agree now but decide to change your mind and withdraw later, the services you receive at the clinic will continue normally.

You will receive different doses of CQ according to your age(FDA approved) over three consecutive days. The medicine, CQ is recommended by the Ministry of Health and this medicine is known to be very effective for *p.vivax*

During the follow-up, a small amount of blood will be taken which is assumed as 7/9 times from your finger. You may experience a bit of pain or fear when your finger is pricked. The pain should disappear within 1 day. The blood will be dropped onto a slide. The blood sample will be used to study the presence of malaria parasite in your blood. Blood sample examination will be done after the treatment and it will not affect the success of the treatment. Nothing else will be done with your blood.

The study will take place for 28 days. During that time, you will have to come to the health facility for 1 hour each day for 7/9 days for the first three days and then once per week for consecutive three weeks. At the end of 1 month, the study will be finished. At each visit, you will be examined by a physician.

Today, I will take and blood for testing. After the tests, you will receive the first dose of treatment if you have the parasite.

On the:

- 2^nd^ visit: you will receive the 2^nd^ dose of treatment plus a blood sample.
- 3^rd^ visit: you will receive the 3rd dose of treatment plus a blood test.
- 4th, 5th, 6th, 7th, and 8th visits, you will have a blood test for parasites and clinical outcome

The medicine may have some unwanted or unexpected effects; however, we will follow you closely and keep track of these effects, if they arise, and of any other problems, you can come to this health facility at any time and ask to see medical seek. If you are experiencing side effects, we may use some other medicine, free of charge, which will help to reduce the symptoms or reactions.

If you decide to participate in this study, any illnesses related to malaria or to the malaria treatment will be treated with no charge to you and your family, and we will cover your transportation expense in relation to the study. Your participation will help us to make sure the medicine is still working, and this will benefit to the society and future generations.

For children younger than the age of 18, parents or guardians will be informed and sign the consent form

Dear participant, I thank you for your cooperation. I would like to ask you to agree and take part in the study by giving the following declaration.

- I have been given detailed information about this study
- I had the opportunity to discuss any question with the researchers
- I understand that my participation in study is voluntary
- I agree to take part in this study

Name of the participant _____________________________ ID No __________________

Signature of the participant or Signature of witness Signature of

Parent/ guardian & date & Date Researcher & date

_______________ _______________ ____________

N.B This Consent from will be translated to local languages for the participants (Amharic)

**Inclusion criteria:**

- Patient’s age ≥ 6 months old
- Uncomplicated *P. vivax* malaria (mono- infection with *P. vivax*)
- Parasite density >250/µ1
- Rectal/ tympanic temperature ≥38 ^0^c or axillary temperature ≥37.5^0^c or recorded history of fever with in the preceding 24 hrs
- Ability to tolerate oral chemotherapy
- Informed consent by the patient or the legal representative
- Resident in the study area

**Exclusion criteria:**

- Adequate anti-malarial treatment within the previous 7 days
- Mixed plasmodium (*P.falciparum* + *p. vivax* or other) infection
- Danger signs (Unable to drink: repeated vomiting: recent history of convulsions: Lethargic or unconscious state: Unable to stand up or to sit) and signs of severe malaria as defined by the WHO.
- Any other known severe underlying disease (e.g. cardiac, renal, hepatic diseases, Malnutrition, known HIV infection)
- Concomitant disease masking assessment response.
- History of allergy or intolerance against study medication
- Pregnancy/Lactation
