# Supplementary material for: Efficacy of Chloroquine for the Treatment of Vivax malaria in Northwest Ethiopia
Source: PLoS One. 2016 Aug 31;11(8):e0161483. doi: 10.1371/journal.pone.0161483 (PMC5007045; doi:10.1371/journal.pone.0161483)
Supplement: S2 File — (DOC) [file pone.0161483.s002.doc]

**6. Questionnaire, case record and screening forms**

**6.1. Screening forms**

**Patient (Code):** ____________________________

**Parent/Guardian (Code):**____________________

Age ___________

ID NO _________

Hospital No _______

Lab. No _____________

**6.1.** **Screening checklist**

**Day 0 (Recruitment)**

Date: _____/______/______ Hospital (Center) No ________

Initials of clinician:__________________ Lab. No _________________

**Patient (Code):** _____________________ Id No ______________

Age: _________ (Years) sex: - Male female

Origin: - Bullen City Outside Bullen

Marital status Single Married Widowed/Divorced

Education: No formal education 1-8^th^ above 9^th^

Ethnicity: Shinasaha Amhara Gumuz Others

| Heamoglobin  level (mg/l) |  | |  | | |
| --- | --- | --- | --- | --- | --- |
| Weight (Kg): |  | |  | | |
| Temperature (^0^C): |  | |  | | |
| Patient history | | | | | |
| Symptoms | | No (please tick) | | Yes (please tick) | Onset of symptoms |
| Fever | |  | |  |  |
| Duration of fever (Hrs) | |  | |  |  |
| Shivering | |  | |  |  |
| Headache | |  | |  |  |
| Nausea | |  | |  |  |
| Diarrhea | |  | |  |  |
| Abdominal pain | |  | |  |  |
| Dizziness | |  | |  |  |
| Vomiting | |  | |  |  |
| Skeletal muscle weakness | |  | |  |  |
| Others (Please specify): | |  | |  |  |

- Does the patient have any other known illnesses?

If yes, please mention: ______________________________________

- Does the patient currently take any medicine regularly?

If yes, please mention: ______________________________________

- Did the patient have malaria/fever?
- Did he/she take any anti-malarial drugs within the last 6 weeks?

If yes, please specify________________________________________________

- Does the patient fulfill all inclusion criteria and no exclusion criteria?

Yes No →Exclusion

- Has the patient or guardian signed the informed consent form?

Yes No

Laboratory results (microscopy):

Thin/thick smear Negative

Positive

P. falciparum P. vivax/ovale P. malariae

Asexual Parasitaemia: __________Gametocytemia_________________

**Day-0 (Recruitment):** _______/_______/______

Day 2: _____/______/_______

Day 3: _____/______/_______

Day 7: _____/______/_______

Day 21:_____/_____/_______

Day 28:_____/_____/_______

**Unscheduled visit:** ____/____/_____

**6.1.** **Case records**

**Day 2 (Follow-up)**

Date:_______/_______/________ Hospital (center) NO. ______

Initials of clinician:______________ Lab. No _________________

Patient (Code): ________ Id No: ______________

Age :__________( years) Sex__________________

| Weight (Kg): |  |  | |
| --- | --- | --- | --- |
| Temperature (^0^C): |  |  | |
| Patient history | | | |
| Symptoms | No (please tick) | Yes (please tick) | Onset of symptoms |
| Fever |  |  |  |
| Shivering |  |  |  |
| Headache |  |  |  |
| Nausea |  |  |  |
| Diarrhea |  |  |  |
| Skeletal muscle weakness |  |  |  |
| Abdominal pain |  |  |  |
| Vomiting |  |  |  |
| Dizziness |  |  |  |
| Others (Please specify): |  |  |  |

- Did the patient take the study medication regularly up to this point in time?

Yes No →Exclusion & Give adequate treatment

If no, explain: _____________________________________________

- Has the patient developed danger signs or sever malaria?

Yes No →ETF, Refer to Hospital to get standard therapy for severe malaria.

- Laboratory results (microscopy):

Thin/thick smear Negative

Positive

P. falciparum P. vivax/ovale P. malariae

- Asexual Parasitaemia: __________ Gametocytemia_______________
- If smear is positive, is the parasitemia higher than on Day 0?

No Yes →ETF, refer the patient to Hospital to get adequate alternative treatment.

**Day 3 (Follow-up)**

Date___/____/________ Hospital (Center) No. __________

Initials of Clinician:_______________ Lab. NO. __________________

Patient (Code): ___________________ ID No: _____________

Age: ___________ (years) Sex__________________

| Weight (Kg): |  |  | |
| --- | --- | --- | --- |
| Temperature (^0^C): |  |  | |
| Patient history | | | |
| Symptoms | No (please tick) | Yes (please tick) | Onset of symptoms |
| Fever |  |  |  |
| Shivering |  |  |  |
| Headache |  |  |  |
| Nausea |  |  |  |
| Skeletal muscle pain |  |  |  |
| Diarrhea |  |  |  |
| Dizziness |  |  |  |
| Vomiting |  |  |  |
| Abdominal pain |  |  |  |
| Others (Please specify): |  |  |  |

- Did the patient finish the study medication properly?

Yes No Exclusion & Adequate treatment

- If No, explain: _________________________________________________
- Has the patient developed danger signs or severe malaria?

No Yes ETF, Refer to Hospital to get standard therapy for severe malaria

- Laboratory results (Microscopy):

Thin/thick smear Negative Positive

P.falciparum P. vivax/ovale P. malariae

Asexual Parasitaemia:_______Gametocytemia_____________

- If positive smear, is the parasitaemia higher than 25% of the count on Day 0?

No Yes ETF, Refer patient to Hospital to get adequate alternative treatment

- If Yes, is the axillaries’ temperature ≥ 37.5 ^o^C? No Yes ETF, Refer to Hospital

**Day 7 (Follow-up)**

Date ___/____/________ Hospital (Center) No. __________

Initials of Clinician: _______________ Lab. NO. __________________

Patient (Code): ___________________ ID No: _____________

Age: ___________ (years) Sex____________________

| Weight (Kg): |  |  | |
| --- | --- | --- | --- |
| Temperature (^0^C): |  |  | |
| Patient history | | | |
| Symptoms | No (please tick) | Yes (please tick) | Onset of symptoms |
| Fever |  |  |  |
| Shivering |  |  |  |
| Vomiting |  |  |  |
| Dizziness |  |  |  |
| Headache |  |  |  |
| Nausea |  |  |  |
| Diarrhea |  |  |  |
| Skeletal muscle weakness |  |  |  |
| Abdominal pain |  |  |  |
| Others (Please specify): |  |  |  |

- Has the patient developed danger signs or sever malaria after Day 3?

No Yes LTF, Refer patient to Hospital to get standard therapy for sever

Malaria Laboratory results (Microscopy):

Thin/thick smear Negative

Positive

P. falciparum P. vivax P. ovale P. malariae

Asexual Parasitaemia:______________Gametocytemia__________________

- If positive smear, is auxiliary’s temperature ≥37.5p C (or History of fever in the last 24 h)?

No → LTF (LPF), Refer to Hospital to get adequate alternative treatment

Yes → LTF (LCF), Refer to Hospital to get adequate alternative treatment

**Day 14 (Follow-up)**

Date: _____/______/______ Hospital (Center) No.__________

Initials of Clinician: __________________ Lab. No. ____________________

Patient (Code):__________________________ ID No: __________

Age :________( Years) Sex_______________

| Weight (Kg): |  |  | |
| --- | --- | --- | --- |
| Temperature (^0^C): |  |  | |
| Patient history | | | |
| Symptoms | No (please tick) | Yes (please tick) | Onset of symptoms |
| Fever |  |  |  |
| Shivering |  |  |  |
| Headache |  |  |  |
| Nausea |  |  |  |
| Diarrhea |  |  |  |
| Skeletal muscle weakness |  |  |  |
| Vomiting |  |  |  |
| Dizziness |  |  |  |
| Abdominal pain |  |  |  |
| Others (Please specify): |  |  |  |

Has the patient developed danger signs or severe malaria after Day-28?

No Yes →LTF, Refer patient to hospital to get standard therapy for severe malaria

Laboratory results (Microscopy):

Thin/thick smear Negative

Positive

P. falciparum P.vivax/ovale P. malaria

Asexual Parasitaemia: __________________Gametocytemia___________

If positive, is axillary temperature $\geq$ 37.5 ^0^C (or history of fever in the last 24h)?

No LTF (LPF), Refer to Hospital to get adequate alternative treatment

Yes LTF (LCF), Refer to Hospital to get adequate alternative treatment

**Day 21 (Follow-up)**

Date___/____/________ Hospital (Center) No. __________

Initials of Clinician:_______________ Lab. NO. __________________

Patient (Code): ___________________ ID No: _____________

Age: ___________ (years) Sex_________________

| Weight (Kg): |  |  | | |
| --- | --- | --- | --- | --- |
| Temperature (^0^C): |  |  | | |
| Patient history | | | | |
| Symptoms | No (please tick) | | Yes (please tick) | Onset of symptoms |
| Fever |  | |  |  |
| Shivering |  | |  |  |
| Headache |  | |  |  |
| Nausea |  | |  |  |
| Skeletal muscle weakness |  | |  |  |
| Vomiting |  | |  |  |
| Dizziness |  | |  |  |
| Diarrhea |  | |  |  |
| Abdominal pain |  | |  |  |
| Others (Please specify): |  | |  |  |

- Has the patient developed danger signs or sever malaria after Day 7?

No Yes LTF, Refer patient to Hospital to get standard therapy for sever malaria

- Laboratory results (Microscopy): Thin/thick smear Positive Negative

P.falciparum P. vivax P. ovale P. malariae

Asexual Parasitaemia:______________Gametocytemia______________

- If positive, is axillary t temperature >37.5^o^C (or History of fever in the last 24 h)?

No → LTF (LPF), Refer to Hospital to get adequate alternative treatment

Yes → LTF (LCF), Refer to Hospital to get adequate alternative treatment

- Laboratory results (Microscopy):

Thin/thick smear Negative Positive

P. falciparum P. vivax P. ovale P. malariae

Asexual Parasitaemia:_____________Gametocytemia_________________

- If positive smear, is the parasitaemia higher than on day-0?

Yes No →ETF, Refer to Hospital to get adequate alternative treatment

**Day 28 (Follow-up)**

Date___/____/________ Hospital (Center) No. __________

Initials of Clinician:_______________ Lab. NO. __________________

Patient (Code):___________________ ID No: _____________

Age: ___________ (years) Sex______________

| Heamoglobin level (mg/l) |  | |  | | |
| --- | --- | --- | --- | --- | --- |
| Weight (Kg): |  | |  | | |
| Temperature (^0^C): |  | |  | | |
| Patient history | | | | | |
| Symptoms | | No (please tick) | | Yes (please tick) | Onset of symptoms |
| Fever | |  | |  |  |
| Shivering | |  | |  |  |
| Headache | |  | |  |  |
| Nausea | |  | |  |  |
| Diarrhea | |  | |  |  |
| Skeletal muscle weakness | |  | |  |  |
| Vomiting | |  | |  |  |
| Dizziness | |  | |  |  |
| Abdominal pain | |  | |  |  |
| Others (Please specify): | |  | |  |  |

- Has the patient developed danger signs or sever malaria after Day 7?

No Yes LTF, Refer patient to Hospital to get standard therapy for sever malaria

- Laboratory results (Microscopy):

Thin/thick smear Negative Positive

P.falciparum P. vivax P. ovale P. malariae

Asexual Parasitaemia:______________Gametocytemia______________

- If positive, is auxiliary temperature >37.5p C (or History of fever in the last 24 h)?

No → LTF (LPF), Refer to Hospital to get adequate alternative treatment

Yes → LTF (LCF), Refer to Hospital to get adequate alternative treatment

- Laboratory results (Microscopy): Thin/thick smear Negative Positive

P. falciparum P. vivax P. ovale P. malariae

Asexual Parasitaemia:___________________Gametocytemia_____________

- If positive smear, is the parasitaemia higher than on day- 0?

Yes No →ETF, Refer to Hospital to get adequate alternative treatment

**6.3 Unscheduled Visit (Follow-up)**

Date: _____/______/______ Hospital (Center) No.__________

Initials of Clinician:__________________ Lab. No. ____________________

Patient (Code):__________________________ ID No: __________

Age :________( Years) Sex__________

| Weight (Kg): |  |  | |
| --- | --- | --- | --- |
| Temperature (^0^C): |  |  | |
| Patient history | | | |
| Symptoms | No (please tick) | Yes (please tick) | Onset of symptoms |
| Fever |  |  |  |
| Shivering |  |  |  |
| Skeletal muscle weakness |  |  |  |
| Dizziness |  |  |  |
| Weakness |  |  |  |
| Headache |  |  |  |
| Nausea |  |  |  |
| Diarrhea |  |  |  |
| Abdominal pain |  |  |  |
| Others (Please specify): |  |  |  |

- Has the patient developed danger signs or severe malaria after Day-28?
- No YesLTF, Refer patient to hospital to get standard therapy for severe malaria
- Laboratory results (Microscopy):

Thin/thick smear Negative

Positive

P. falciparum P.vivax/ovale P. malaria

Asexual Parasitaemia: __________________Gametocytemia

- If positive, is axillary temperature $\geq$ 37.5 ^0^C (or history of fever in the last 24h)?

No LTF (LPF), Refer to Hospital to get adequate alternative treatment

Yes LTF (LCF), Refer to Hospital to get adequate alternative treatment

**6.3** **Drop out Form**

Date: _____/______/______ Hospital (Center) No.__________

Initials of Clinician:__________________ Lab. No. ____________________

**Patient (Code):**__________________________ ID No: __________

**Screening Failure**

Reason:________________________________________

**Treatment Failure:-**

**ETF (Early Treatment Failure)** ▫

**LCF (Late Clinical Failure)**▫

**LPF (Late Parasitological Failure)**▫

Day: ________________________

Explanation: ____________________________________________________

Lost to Follow–up:

Explanation: - _________________________________________________

Withdrawal:

Reason: - _____________________________________________________

Protocol Violation

Explanation: - __________________________________________________
