# Supplementary figures and images for: Efficacy of Chloroquine for the Treatment of Vivax malaria in Northwest Ethiopia
Source: PLoS One. 2016 Aug 31;11(8):e0161483. doi: 10.1371/journal.pone.0161483 (PMC5007045; doi:10.1371/journal.pone.0161483)

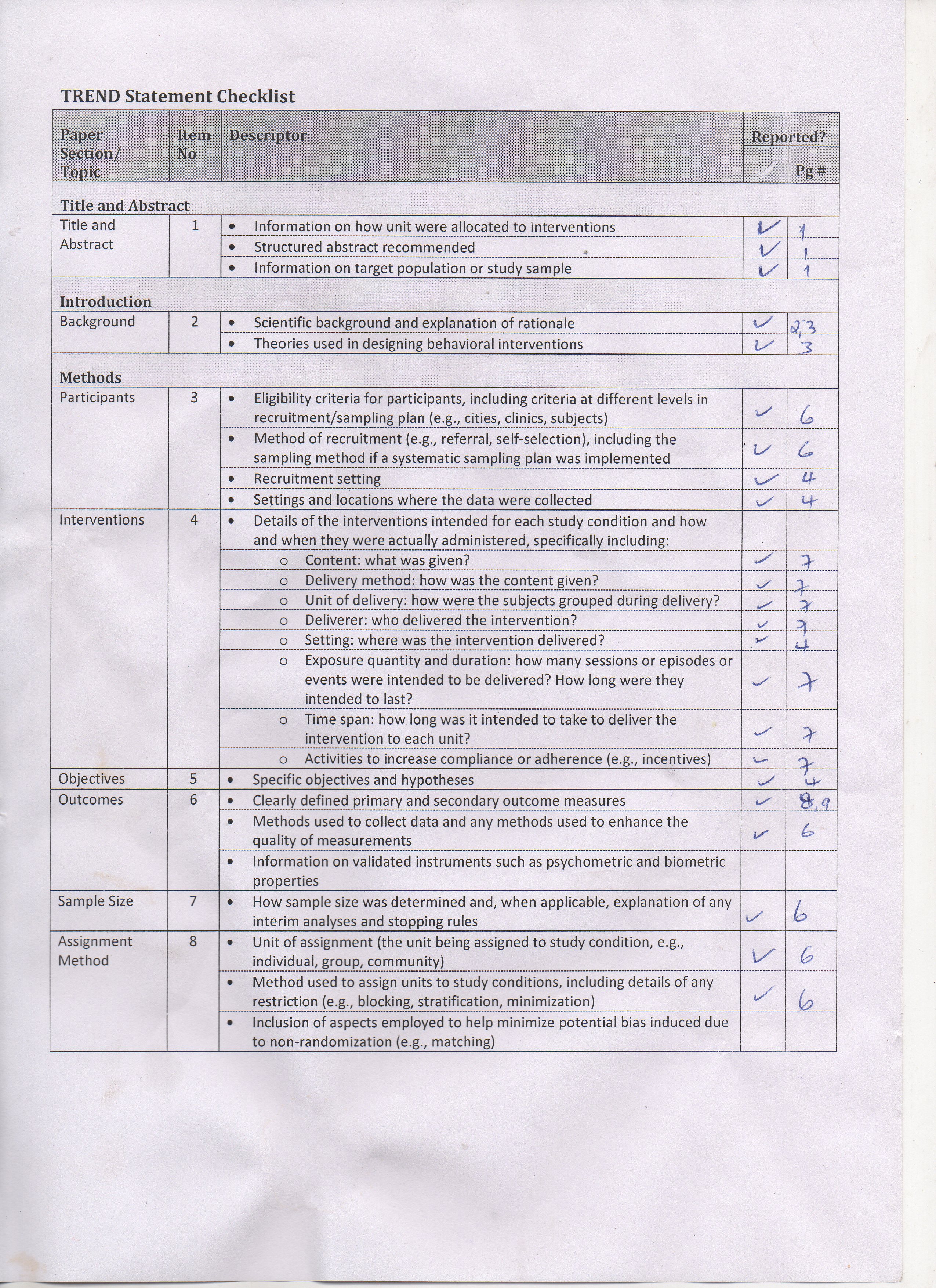


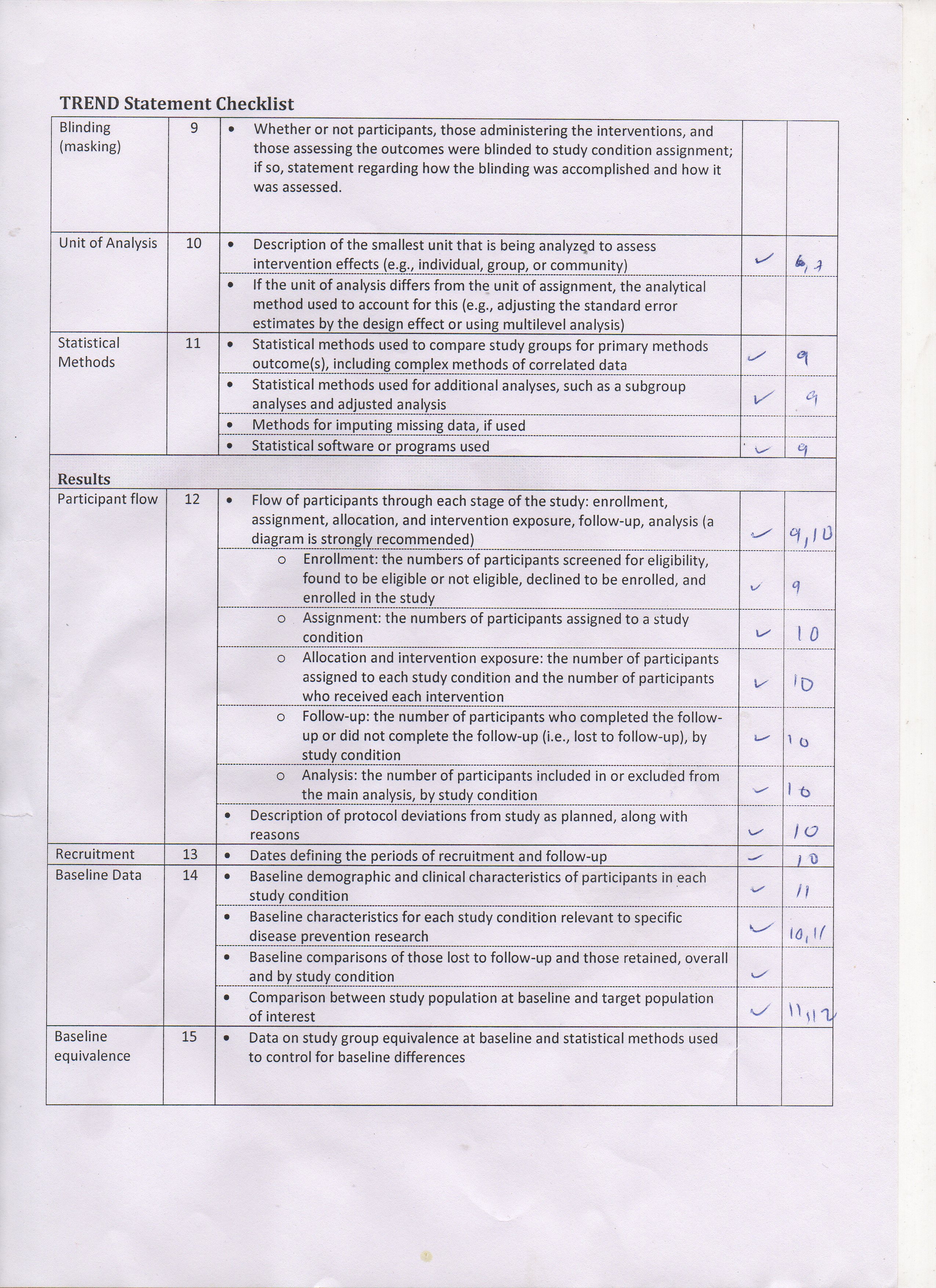


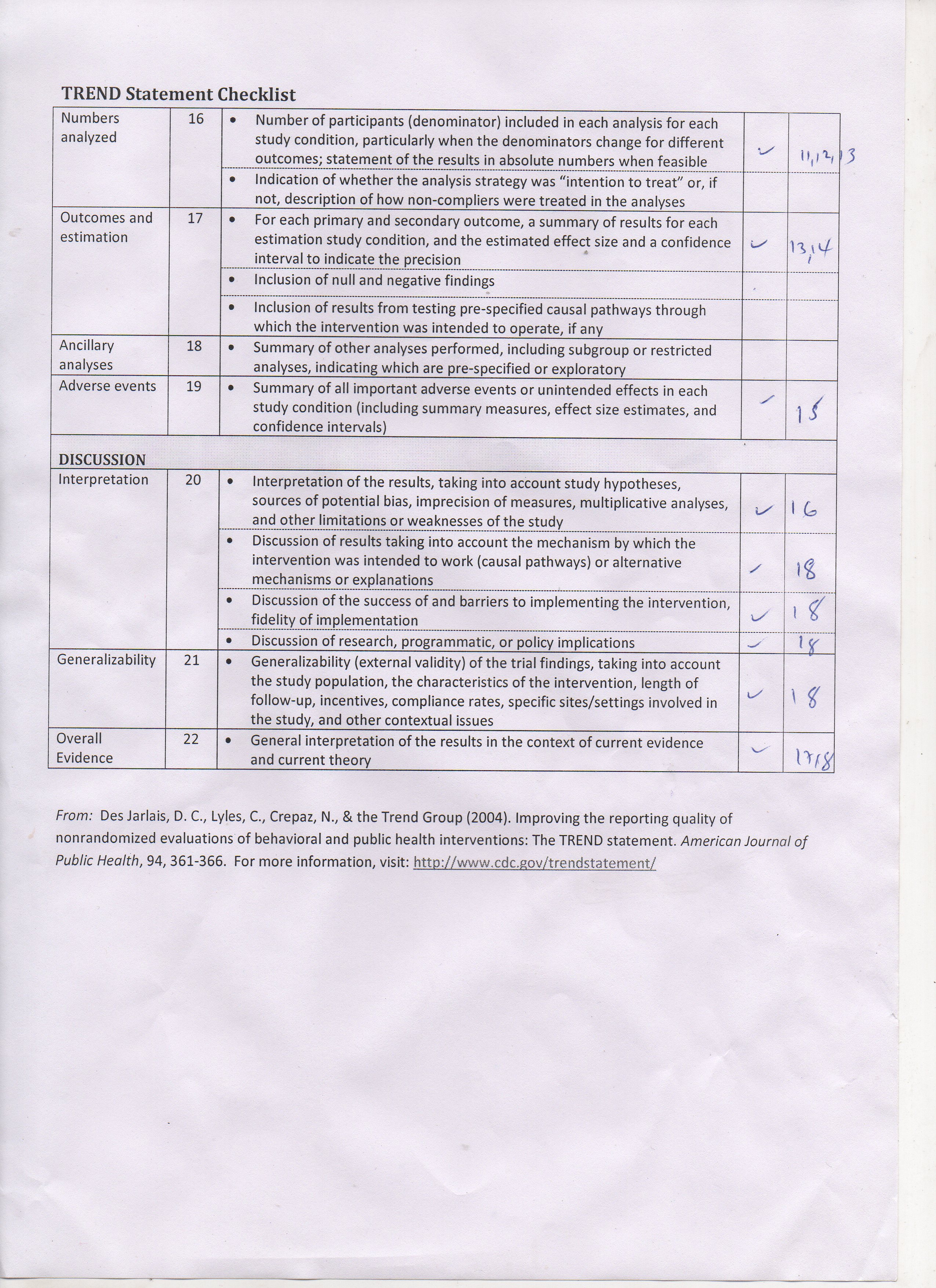

Supplement: S3 File — (DOC) [file pone.0161483.s003.doc]
